# Systematic characterization of full-length RNA isoforms in human colorectal cancer at single-cell resolution

**DOI:** 10.1093/procel/pwaf049

**Published:** 2025-07-22

**Authors:** Ping Lu, Yu Zhang, Yueli Cui, Yuhan Liao, Zhenyu Liu, Zhi-Jie Cao, Jun-e Liu, Lu Wen, Xin Zhou, Wei Fu, Fuchou Tang

**Affiliations:** Biomedical Pioneering Innovative Center, School of Life Sciences, Department of General Surgery, Third Hospital, Peking University, Beijing 100871, China; Beijing Advanced Innovation Center for Genomics (ICG), Ministry of Education Key Laboratory of Cell Proliferation and Differentiation, Beijing 100871, China; Changping Laboratory, Beijing 102206, China; Biomedical Pioneering Innovative Center, School of Life Sciences, Department of General Surgery, Third Hospital, Peking University, Beijing 100871, China; Beijing Advanced Innovation Center for Genomics (ICG), Ministry of Education Key Laboratory of Cell Proliferation and Differentiation, Beijing 100871, China; Changping Laboratory, Beijing 102206, China; Biomedical Pioneering Innovative Center, School of Life Sciences, Department of General Surgery, Third Hospital, Peking University, Beijing 100871, China; Beijing Advanced Innovation Center for Genomics (ICG), Ministry of Education Key Laboratory of Cell Proliferation and Differentiation, Beijing 100871, China; Biomedical Pioneering Innovative Center, School of Life Sciences, Department of General Surgery, Third Hospital, Peking University, Beijing 100871, China; Beijing Advanced Innovation Center for Genomics (ICG), Ministry of Education Key Laboratory of Cell Proliferation and Differentiation, Beijing 100871, China; Biomedical Pioneering Innovative Center, School of Life Sciences, Department of General Surgery, Third Hospital, Peking University, Beijing 100871, China; Beijing Advanced Innovation Center for Genomics (ICG), Ministry of Education Key Laboratory of Cell Proliferation and Differentiation, Beijing 100871, China; Changping Laboratory, Beijing 102206, China; Biomedical Pioneering Innovative Center, School of Life Sciences, Department of General Surgery, Third Hospital, Peking University, Beijing 100871, China; Beijing Advanced Innovation Center for Genomics (ICG), Ministry of Education Key Laboratory of Cell Proliferation and Differentiation, Beijing 100871, China; Changping Laboratory, Beijing 102206, China; Biomedical Pioneering Innovative Center, School of Life Sciences, Department of General Surgery, Third Hospital, Peking University, Beijing 100871, China; Beijing Advanced Innovation Center for Genomics (ICG), Ministry of Education Key Laboratory of Cell Proliferation and Differentiation, Beijing 100871, China; Changping Laboratory, Beijing 102206, China; Biomedical Pioneering Innovative Center, School of Life Sciences, Department of General Surgery, Third Hospital, Peking University, Beijing 100871, China; Beijing Advanced Innovation Center for Genomics (ICG), Ministry of Education Key Laboratory of Cell Proliferation and Differentiation, Beijing 100871, China; Changping Laboratory, Beijing 102206, China; Biomedical Pioneering Innovative Center, School of Life Sciences, Department of General Surgery, Third Hospital, Peking University, Beijing 100871, China; Peking University Third Hospital Cancer Center, Beijing 100191, China; Beijing Key Laboratory of Collaborative Innovation in Gastrointestinal Oncology, Beijing 100191, China; Biomedical Pioneering Innovative Center, School of Life Sciences, Department of General Surgery, Third Hospital, Peking University, Beijing 100871, China; Peking University Third Hospital Cancer Center, Beijing 100191, China; Beijing Key Laboratory of Collaborative Innovation in Gastrointestinal Oncology, Beijing 100191, China; Biomedical Pioneering Innovative Center, School of Life Sciences, Department of General Surgery, Third Hospital, Peking University, Beijing 100871, China; Beijing Advanced Innovation Center for Genomics (ICG), Ministry of Education Key Laboratory of Cell Proliferation and Differentiation, Beijing 100871, China; Changping Laboratory, Beijing 102206, China; Peking-Tsinghua Center for Life Sciences, Academy for Advanced Interdisciplinary Studies, Peking University, Beijing 100871, China

**Keywords:** colorectal cancer, long-read scRNA-seq, full-length RNA isoforms, RNA splicing, differential transcript usage

## Abstract

Dysregulated RNA splicing is a well-recognized characteristic of colorectal cancer (CRC); however, its intricacies remain obscure, partly due to challenges in profiling full-length transcript variants at the single-cell level. Here, we employ high-depth long-read scRNA-seq to define the full-length transcriptome of colorectal epithelial cells in 12 CRC patients, revealing extensive isoform diversities and splicing alterations. Cancer cells exhibited increased transcript complexity, with widespread 3′-UTR shortening and reduced intron retention. Distinct splicing regulation patterns were observed between intrinsic-consensus molecular subtypes (iCMS), with iCMS3 displaying even higher splicing factor activities and more pronounced 3′-UTR shortening. Furthermore, we revealed substantial shifts in isoform usage that result in alterations of protein sequences from the same gene with distinct carcinogenic effects during tumorigenesis of CRC. Allele-specific expression analysis revealed dominant mutant allele expression in key oncogenes and tumor suppressors. Moreover, mutated *PPIG* was linked to widespread splicing dysregulation, and functional validation experiments confirmed its critical role in modulating RNA splicing and tumor-associated processes. Our findings highlight the transcriptomic plasticity in CRC and suggest novel candidate targets for splicing-based therapeutic strategies.

## Introduction

RNA splicing is finely regulated to generate diverse mature transcript variants to expand the proteomic repertoire (Blencowe, 2006; Wang et al., 2008; Wright et al., 2022). During this process, exons and introns are selectively included or excluded, which is called alternative splicing (AS). Aberrant splicing regulation resulting from abnormal expression and recurrent mutations of trans- or cis- regulators, as well as aberrant alternative splicing patterns, can promote tumorigenesis through mechanisms of excessive cell proliferation, diminished apoptosis, invasion and metastasis, and resistance to therapy (Bonnal et al., 2020; Bradley and Anczukow, 2023; Sveen et al., 2016). An archetypal example is the switch of alternative 5′ splice site in exon 2 of *BCL2L1*, generating functionally distinct RNA and protein isoforms BCL-*x*L and BCL-*x*S that are anti- and pro-apoptotic, respectively, with the former overexpressed in many types of cancers to promote tumor progression, including breast cancer, non-small cell lung cancer and prostatic cancer (Boise et al., 1993; Castilla et al., 2006; Espana et al., 2004; Ikegaki et al., 1994; Trisciuoglio et al., 2017). In addition, serine/arginine-rich (SR) protein family members (e.g., SRSF1, SRSF3 and SRSF4) and heterogeneous nuclear ribonucleoprotein (hnRNP) protein family members (e.g., hnRNPA1 and hnRNPA2/B1) frequently regulate alternative splicing in a concentration-dependent manner (Anczukow et al., 2015; Bradley and Anczukow, 2023; Geuens et al., 2016; Jia et al., 2019; Lee and Rio, 2015; Urbanski et al., 2018). Despite that scattering critical splicing regulators and alternative splicing isoforms have been characterized in cancer, the underlying mechanisms involved in post-transcriptional regulation are still not well-explored, underscoring the urgent need for systematic decipherment of full-length transcriptome.

Studies of colorectal cancer (CRC) transcriptomes have focused on either intratumoral heterogeneities of gene expression using short-read single-cell RNA-seq (scRNA-seq) (Dai et al., 2019; Joanito et al., 2022; Li et al., 2017; Pelka et al., 2021; Smillie et al., 2019) or RNA splicing patterns using bulk RNA-seq (Kahles et al., 2018; Liu et al., 2018; Xiong et al., 2018; Xu et al., 2022; Zong et al., 2018). To date, the elusive combination of single-cell resolution and full-length transcript coverage has not been attained in prior attempts to profile RNA isoform heterogeneities and diversities in colorectal cancer. Recently, long-read scRNA-seq has emerged as a powerful solution to overcome the limitations of short-read assembly discontinuities, and to simultaneously identify full-length isoforms, splice junctions (SJs), poly(A) tail length, etc. (Amarasinghe et al., 2020; Byrne et al., 2017, 2019; Fan et al., 2020; Gupta et al., 2018; Joglekar et al., 2021; Lebrigand et al., 2020; Philpott et al., 2021; Sakamoto et al., 2020; Singh et al., 2019; Stark et al., 2019; Tian et al., 2021; You et al., 2023).

To this end, we focused on the obscured intricacies of the full-length transcriptome. We employed the SCAN-seq (single-cell amplification and sequencing of full-length RNAs by Nanopore platform) method (Fan et al., 2020) and its more scalable version, SCAN-seq2 (Liao et al., 2023). The former provides data with high depth, while the latter increases throughput, enhancing the robustness of our results. Using these methods, we generated high-fidelity single-cell full-length transcriptomic profiles from primary tumors and matched normal controls in 12 colorectal cancer patients. Unlike prior studies, our data enabled a nuanced analysis of isoform diversity in colorectal cancer *in vivo*, alongside an exploration of RNA splicing regulatory mechanisms. We identified variations in UTR lengths and alternative splicing events, elucidating their regulatory interplay with splicing factors (SFs). Linking somatic mutations of corresponding CRC patients from whole-exome sequencing, we discerned the transcriptional phenotypic impacts of allele-specific expression patterns. Significantly, we revealed isoform switches that lead to protein alterations in key genes implicated in tumorigenesis. This comprehensive resource offers novel insights and potential targets for the clinical diagnosis and treatment of colorectal cancer, thereby laying the groundwork for more efficacious treatment modalities.

## Results

### Profiling cellular heterogeneities of CRC at the individual RNA isoform level

**Figure 1.**
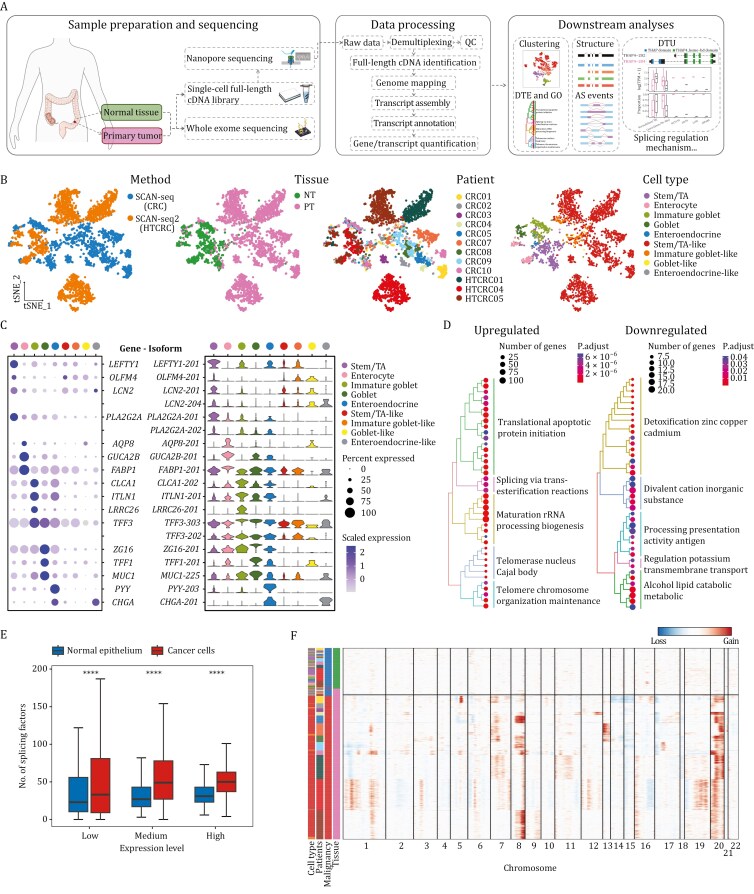
Single-cell full-length transcriptomic atlas of CRC. (A) Workflow Overview. An illustrative schematic detailing the process from sample preparation through sequencing and data analysis. (B) t-SNE plots of cells colored by method (SCAN-seq for CRC data, SCAN-seq2 for HTCRC data), tissue type (Normal Tissue, NT; Primary Tumor, PT), patient ID and cell type, from left to right. (C) The expression of cell type specific marker genes (dot plot) and corresponding isoforms (violin plot) across cell types. (D) Hierarchical clustering of top 25 enriched GO terms for genes associated with upregulated (left) and downregulated (right) isoforms in cancer cells. (E) Box plot showing the number of splicing factors grouped by low, medium, and high expression levels in normal epithelial cells versus cancer cells. Statistical significance was determined using the two-tailed Wilcoxon rank-sum test, with ****P < 0.0001. (F) Heatmap showing large-scale chromosomal CNAs of cells inferred using normal tissue cells as reference. Each row represents a cell, which is color-labeled as in panels (B) and (E).

To investigate the heterogeneities of full-length transcriptome in CRC, we performed long-read scRNA-seq on 5,400 CD45^−^EPCAM^+^ cells isolated from primary tumors (PT) and adjacent normal tissues (NT) of 12 CRC patients. After rigorous quality control and filtering (Methods), we retained 3,262 high-quality cells, comprising 1,401 cells from SCAN-seq and 1,861 cells from SCAN-seq2 (Figs. 1A, 1B, S1A; Table S1 and Methods). Overall, 29,429 non-redundant transcript isoforms from 12,827 genes were obtained after genome mapping, transcript assembly, correction, and filtering (Methods). For SCAN-seq data, the average number and length of mapped full-length cDNA reads per cell were 322,496 and 1,112 bp, respectively, with an average of 3,897 genes and 5,760 isoforms detected per cell (Fig. S1B–E), signifying a high-precision resource and a substantial prerequisite to mine transcript diversities and underlying splicing mechanisms at the single-cell level. For SCAN-seq2 data, the average number and length of mapped full-length cDNA reads per cell was 17,826 and 717 bp, respectively, with an average of 1,987 genes and 2,664 isoforms detected per cell, offering complementary insights for constructing a comprehensive single-cell transcriptomic landscape.

Dimension reduction and clustering analyses based on isoform count matrix categorized these cells into 798 normal epithelial cells and 2,464 malignant cancer cells, with the former further refined into stem/transit-amplifying (stem/TA) cells (*LEFTY1*, *OLFM4*, *LCN2*, *PLA2G2A,* and *SOX9*), enterocytes (*AQP8*, *GUCA2B*, *FABP1*, *CLCA4,* and *SLC26A2*), immature goblet cells (*CLCA1*, *ITLN1*, *LRRC26*, *TFF3,* and *SPINK4*), goblet cells (*ZG16*, *TFF1*, *MUC2*, *MUC1,* and *BCAS1*), and enteroendocrine cells (*PYY*, *CHGA*, *CHGB,* and *REG4*) (Chen et al., 2021a; Gao et al., 2018; Pelka et al., 2021; Wang et al., 2020), and the latter further annotated as stem/TA-like cells, immature goblet-like cells, goblet-like cells and enteroendocrine-like cells by reference component analysis (RCA) (Li et al., 2017) using the normal epithelial cell types as reference (Figs. 1B, 1C, S1F–H; Table S2 and Methods). To ensure the reliability of cell type identification, we integrated our data with the nearly 168,000 epithelial cells of the Human Colon Cancer Atlas (c295) (Pelka et al., 2021). To mitigate the potential impact of cell number differences compared to our dataset, we randomly partitioned the c295 dataset into 5 cohorts with equal cell numbers. All cell types, from both normal and cancer epithelium, were well matched with the consistent identity of the c295 dataset in every reproducible integration (Fig. S1I and Methods).

Of note, cell type-specific markers such as *LCN2*, *PLA2G2A* and *TFF3* express more than one isoform, highlighting the coarse nature of gene-level relative to RNA isoform-level characterization, and the non-negligible functions of transcript variants (Fig. 1C). Benefiting from the isoform-level distinguishability of long read sequencing, we identified 1,137 significantly upregulated isoforms and 271 downregulated isoforms in cancer cells. Programs strongly enriched in cancer cells included splicing via transesterification reactions, positive Cajal body region and ncRNA processing metabolic process (Fig. 1D and Methods), which are closely involved in RNA splicing machinery including ribonucleoprotein (RNP) formation (Liu et al., 2021; Love et al., 2017). Notably, the expression levels of splicing factor genes (as defined by GO annotations ‘RNA splicing’ and ‘spliceosomal complex’) increased in cancer cells, confirming the greater activity of splicing alteration characteristic of CRC transcriptomes (Fig. 1E). Additionally, we inferred large-scale chromosomal copy number alterations (CNAs) of cells using normal cells as reference (Fig. 1F). The result mainly detected copy number gains of 7q, 8q, 13q, and 20q, as well as copy number losses of 14q, 15q, and 18q (Cancer Genome Atlas Network, 2012; Mamlouk et al., 2017), reflecting the heterogeneities among patients, consistent with the cell clustering results described above.

### Systematic characterization of full-length transcriptome complexity in CRC

**Figure 2.**
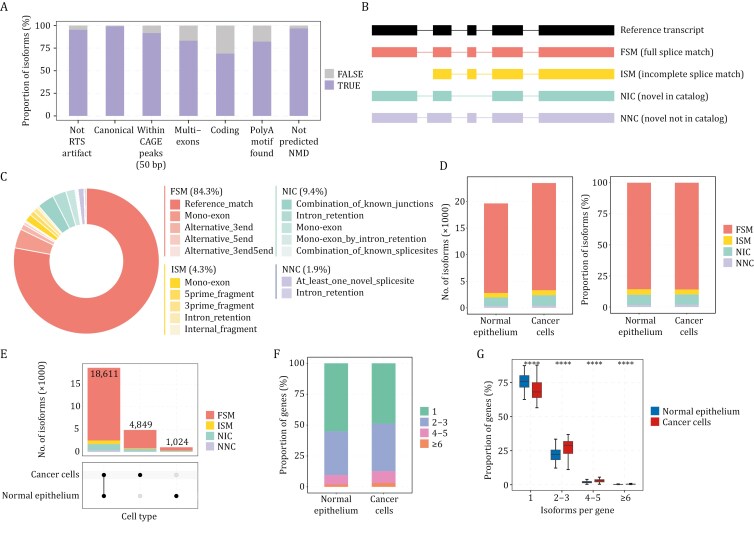
Characterization of the assembled full-length transcriptome in CRC. (A) Quality control metric for the annotated isoforms. (B) Classification of transcript isoforms based on the splice junctions matching with reference transcripts. Diagram illustrating the classification of isoforms based on splice junction matches to the reference transcriptome, categorized into Full Splice Match (FSM), Incomplete Splice Match (ISM), Novel In Catalog (NIC), and Novel Not in Catalog (NNC). (C) Donut chart displaying the distribution of isoform categories and subcategories. (D) Bar plots presenting the total number of (left) and the percentage of (right) isoform categories in normal epithelium and cancer cells. (E) UpSet plot showing the number of isoforms shared between cancer cells and normal epithelium. (F) Stacked bar plot illustrating the distribution of genes grouped by the number of expressed isoforms (1, 2–3, 4–5, and ≥ 6) in normal epithelial and cancer cells. (G) Box plot showing the percentage of genes with varying isoform counts in normal epithelium and cancer cells. Statistical significance was determined using the two-tailed Wilcoxon rank-sum test, with ****P < 0.0001.

To explore the extent of transcriptome complexity in CRC, we characterized the splice junctions and structures of the full-length isoforms. The reliability and accuracy of the assembled transcriptome were first substantiated by quality indications using SQANTI3 (Tardaguila et al., 2018), including reverse transcriptase template switching (RTS) artifacts, canonical/non-canonical splicing junctions, overlap status with CAGE (Cap Analysis Gene Expression) peak and poly(A) tails (Fig. 2A and Methods). Based on the splice junctions matching with reference transcripts by SQANTI3, the 29,429 isoforms were further classified into 4 categories: Full Splice Match (FSM), known transcripts matching all SJs perfectly; Incomplete Splice Match (ISM), known transcripts matching SJs partially; Novel In Catalog (NIC), novel transcripts with a new combination of known donor/acceptor sites; Novel Not in Catalog (NNC), novel transcripts with at least one new splice site, accounting for 84.3%, 4.3%, 9.4%, and 1.9% of the identified isoforms, respectively (Fig. 2B, 2C; Table S3 and Methods).

Next focusing on the differences in transcriptomic feature, we observed that cancer cells expressed higher numbers of both RNA isoforms (6,427 vs. 3,966 isoforms per cell) and genes (4,283 vs. 2,861 genes per cell) than normal epithelium. Moreover, the same trends were preserved after down-sampling by the same cell number and sequencing depth (Fig. S2A). Due to strict filtering to retain the most accurate isoforms, the fractions of FSM isoforms in both normal epithelium and cancer cells were very high. The fraction of novel transcripts was slightly higher in cancer cells (10.1%) than in normal epithelial cells (9.9%) (Fig. 2D), the difference of which was not as large as in other cancer-related long-read RNA-seq datasets (Huang et al., 2021; Veiga et al., 2022). However, we did observe that cancer cells expressed a greater variety of isoforms, indicating a greater number of specific alternative splicing events to generate higher transcript diversities in cancer cells (Fig. 2E). The same conclusion was confirmed by the distribution of RNA isoform expression within the same gene. 51.1% of genes in cancer cells expressed more than one isoform, which was 6.2% higher than in normal epithelium (44.9%) (Fig. 2F). Within each gene, the expression of isoforms displayed a distinct bias, with the most abundant two isoforms collectively accounting for over 90% of the expression (Fig. 2G). These results were further substantiated through down-sampling analysis (Fig. S2B–I).

### APA leads to shorter 3′-UTRs preferentially in stem/TA-like cancer cells

**Figure 3.**
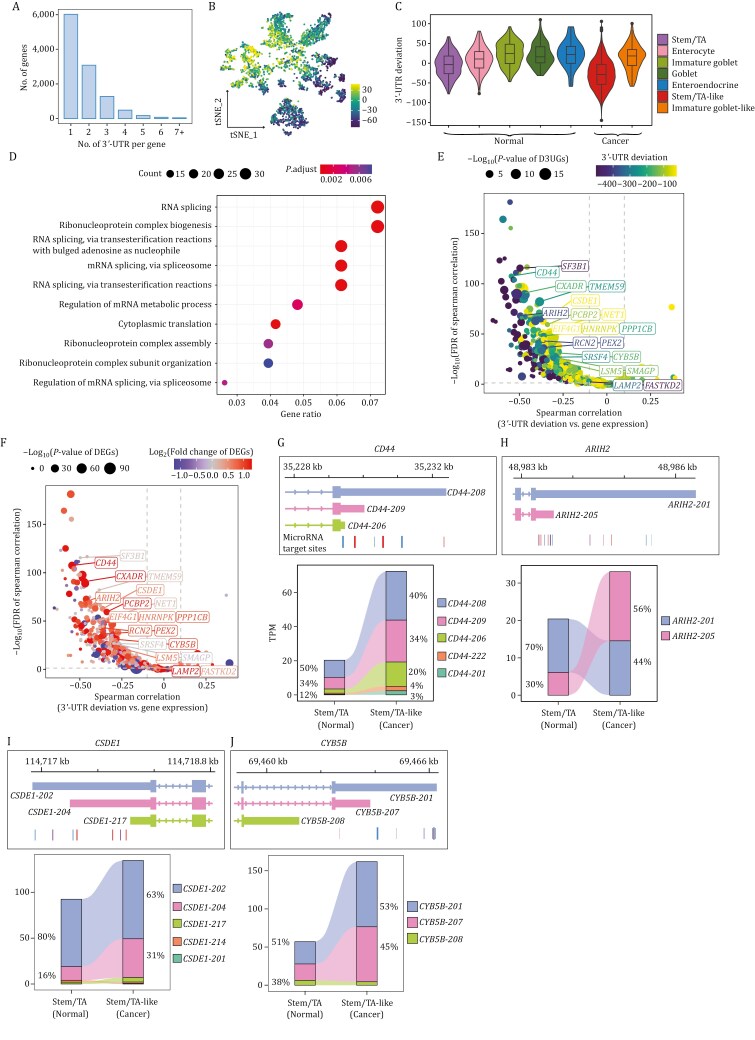
**Analysis of 3**′**-UTR length variations and regulation on gene expression.** (A) Histogram showing the distribution of the number of 3′-UTRs per gene. (B) t-SNE visualization of the mean 3′-UTR deviation of all genes across individual cells. Each point represents a single cell, with the color scale indicating the extent of 3′-UTR length deviation (yellow, shortening; blue, elongation). (C) Violin plot of 3′-UTR deviation across different cell types in CRC. (D) Bubble plot displaying top 10 GO terms enriched for genes with significantly shortened 3′-UTR in cancer cells. The color represents significance and the size represents the number of genes contained in the GO terms. (E and F) Volcano plots of spearman correlation between 3′-UTR deviation and gene expression in significant 3′-UTR shortened genes. In panel (E), the dot color represents 3′-UTR deviation difference between stem/TA-like cells and stem/TA cells, and the dot size represents false discovery rate (FDR) value of spearman correlation. In panel (F), the dot color represents the log_2_(fold change of DEGs) between stem/TA-like cells and stem/TA cells, and the dot size represents the significance of DEGs. (G–J) Representation of isoform structures, microRNA binding sites (top), and corresponding expression levels (bottom) for representative genes with significant 3′-UTR shortening: (G) *CD44*, (H) *ARIH2*, (I) *CSDE1* and (J) *CYB5B*.

Structure annotations and quantifications of alternative RNA isoforms within a gene, which involve different choices of sequence elements including untranslated regions (UTRs), lay the foundation for interpreting post-transcriptional regulation of mRNA stability and translational efficiency. In our long-read scRNA-seq data, about 46.0% and 48.1% of genes were found to express two or more 3′-UTRs and 5′-UTRs, respectively (Figs. 3A and S3A). To explore UTR alteration in colorectal cancer, we defined gene UTR deviation (Methods) measuring the changes in UTR length in each cell compared to normal epithelium as the baseline. Specifically, we first computed the expressed UTR length for each gene in each cell by averaging the UTR lengths of different isoforms weighted by their respective expression (Agarwal et al., 2021). Gene UTR deviation of each cell was then calculated by subtracting the mean expressed UTR length in normal epithelium.

We projected mean 3′-UTR deviation across all genes onto the t-SNE map and observed a global shortening of 3′-UTRs in cancer cells (Fig. 3B), which was mostly enriched in stem/TA-like cells instead of immature goblet-like cells (Fig. 3C). Similarly, cancer cells exhibited 5′-UTR shortening, although less pronounced than the shortening observed in the 3′-UTR, with a notable enrichment in stem/TA-like cancer cells (Fig. S3B and S3C). Thereupon, we sought differential 3′-UTR genes between stem/TA-like (cancer) and stem/TA (normal) cell types and identified 542 3′-UTR shortened genes and 220 3′-UTR lengthened genes (Fig. S3D; Table S4, and Methods). The former was significantly enriched in RNA splicing-related programs and ribonucleoprotein complex biogenesis, while the latter showed little to no significant enrichment in GO terms (Fig. 3D). In addition, we conducted an analysis to calculate the correlation between 3′-UTR deviation and the expression of cleavage and polyadenylation (CPA) regulators, as previously summarized in research (Gruber and Zavolan, 2019; Kowalski et al., 2023). Notably, *SRSF3*, *CPSF3*, *FIP1L1*, *CTR9*, *NUDT21*, and *CSTF2*, among others, were found to exhibit a more significant contribution to the differential 3′-UTR gene expression in colorectal cancer (Fig. S3E). For 5′-UTR, despite the identification of 476 genes with shortened 5′-UTRs and 186 with lengthened 5′-UTRs, none exhibited enrichment in GO terms (Table S4).

To evaluate the regulatory effects of 3′-UTR variation on gene expression, we conducted a correlation analysis between UTR deviation and expression levels for 3′-UTR shortened genes and lengthened genes, respectively. In the case of genes with shortened 3′-UTRs, an overwhelmingly clear correlation was observed between 3′-UTR length shortening and an increase in RNA abundance of the corresponding genes (Fig. 3E and 3F). For example, *CD44* (Chen et al., 2018), *ARIH2* (Geng et al., 2022), *CSDE1* (also known as *UNR*) (Wurth et al., 2016), *CYB5B* (Murphy et al., 2010), *SF3B1* (Alors-Perez et al., 2021), and *RCN2* (Ding et al., 2017; Ning et al., 2023), which have been reported to be overexpressed in cancer, showed an increased proportion of shorter 3′-UTRs, primarily as a result of alternative cleavage and polyadenylation (APA) (Fig. 3G–J). These shorter 3′-UTRs were associated with a reduction in microRNA binding sites, as shown by the microRNA target site predictions from TargetScan database (McGeary et al., 2019) (Fig. 3G–J), which potentially enhance mRNA stability and translational efficiency (Mayr and Bartel, 2009; O’Brien et al., 2018). This result suggests a more intricate regulation of gene expression than previously recognized. For the genes with lengthened 3′-UTRs, no one-sided correlation was observed (Fig. S3F and S3G). Similarly, for genes with shortened or lengthened 5′-UTRs, we observed trends akin to those seen with 3′-UTR variation, albeit with a less pronounced effect on gene expression (Fig. S3H and S3I). These findings reveal that 3′-UTR shortening is a prominent feature in colorectal cancer, likely promoting gene overexpression by mitigating microRNA-mediated regulation.

### Splicing regulation and dynamics in colorectal cancer

**Figure 4.**
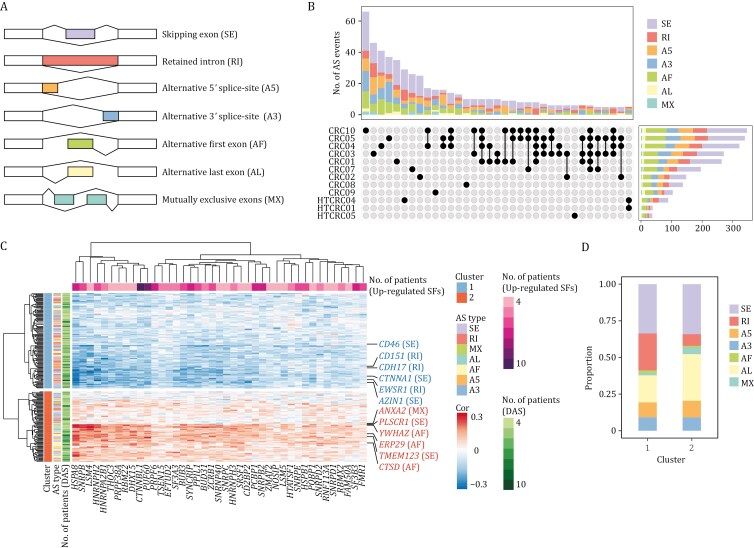
Alternative splicing characterization and their potential modulation by splicing factors. (A) Schematic representation of alternative splicing event types, including skipping exon (SE), retained intron (RI), alternative 5′ splice-site (A5), alternative 3′ splice-site (A3), alternative first exon (AF), alternative last exon (AL), and mutually exclusive exons (MX). (B) UpSet plot displaying the intersection of differential AS events between stem/TA-like cells and stem/TA cells across patients, with bar plots showing the distribution of AS types within each dataset. (C) Heatmap of Pearson correlation coefficients between percent spliced in (PSI) matrices of differential AS events and the expression of differentially expressed splicing factors. (D) Bar plot showing the proportion of AS events in clusters corresponding panel (C).

Building on the established expression profiles of splicing factors and the comprehensive catalog of full-length transcripts, we delved deeper into the alternative splicing events and their potential modulation by SFs in colorectal cancer. Alternative splicing can be divided into 6 types including skipping exon (SE), retained intron (RI), alternative 5′ splice-site (A5), alternative 3′ splice-site (A3), alternative first exon (AF), alternative last exon (AL), mutually exclusive exons (MX) (Fig. 4A). We conducted differential alternative splicing (DAS) analysis using the SUPPA method (Trincado et al., 2018) with criteria set at *P*-value < 0.05 and the difference in percent spliced in (ΔPSI) ≥ 10% between stem/TA-like (cancer) and stem/TA (normal) cell types in each patient (Fig. S4; Table S5 and Methods). A total of 889 unique DAS events were identified, of which SE predominated, followed by AL, RI, A5, and A3 (Fig. S5A). Notably, stem/TA-like cancer cells demonstrated a marked inclination towards diminished intron retention (Fig. S5B). The UpSet plot further illustrates a substantial degree of inter-patient heterogeneities in alternative splicing patterns, underscoring the variability in splicing regulation across different patients (Fig. 4B). Genes with shared AS events in at least three patients were enriched in biological processes such as RNA processing, response to wounding, cell cycle regulation and cellular motility, suggesting their potential common oncogenic roles in tumor progression (Fig. S5C).

To explore the regulation between splicing factors and alternative splicing, we calculated Pearson correlation coefficients between percent spliced in (PSI) matrices of these DAS events and gene expression matrix of up-regulated splicing factors in stem/TA-like cells (cancer) compared with stem/TA cells (normal) (Fig. 4C). Hierarchical clustering analysis classified these DAS events into two distinct clusters. Notably, clusters 1 and 2 exhibited negative and positive correlations with splicing factor expression, respectively. Cluster 1 was significantly enriched in intron retention events, whereas cluster 2 showed a notable depletion of such events (Fisher’s Exact Test, *P*-value < 1.57 × 10^−3^, Fig. 4D). Among the most strongly correlated SFs are HSPA8, a key member of the heat shock protein 70 kDa (HSP70) family; SNRPB, a core component of small nuclear ribonucleoproteins (snRNPs) within the spliceosome; LSM4, an essential part of the LSM2-8 complex which associates with U6 small nuclear RNA (snRNA); and members of the heterogeneous nuclear ribonucleoprotein (hnRNP) family, specifically HNRNPH2 and HNRNPA2B1. These splicing factors demonstrated positive correlations with the differential alternative splicing of genes *ANXA2*, *PLSCR1*, *YWHAZ*, *ERP29*, *TMEM123,* and *CTSD*. In contrast, they showed negative correlations with the alternative splicing of *AZIN1*, *EWSR1*, *CTNNA1*, *CDH17*, *CD151,* and *CD46* (Fig. 4C). These specific SF-AS regulatory programs could potentially play pivotal functional roles in the onset and progression in colorectal cancer.

Next, we explored the dynamic regulation of alternative splicing during CRC progression. The inferred pseudotime trajectory revealed three distinct states, reflecting the differentiation continuum of epithelial cells in CRC, as exemplified by patient CRC01 (Fig. S6A). The trajectory structure suggests a branched differentiation process, with State 1 representing an early/pre-branch stage (corresponding to normal stem/TA cells), which subsequently bifurcates into State 2 (differentiated normal epithelium) and State 3 (cancer cells). Notably, the number of mutations inferred from long-read scRNA-seq per cell increased along pseudotime in the cancer cell trajectory, indicating a progressive accumulation of genetic alterations and increased malignancy during tumor evolution within the same patient.

We analyzed differentially expressed splicing factors across pseudotime states (Fig. S6B). Notably, State 3 (cancer cells) exhibited a greater number of upregulated splicing factors compared to the normal epithelium, indicating an enhanced splicing regulatory activity along the tumor progression trajectory. Key upregulated factors included U4/U6 snRNP components *PRPF3* and *PRPF4*, U2 snRNP-associated genes *SF3B2*, *DHX15*, *HTATSF1*, *SF3A3*, and *PUF60*, C complex factors *PPWD1*, *DDX41*, and *DHX38*, as well as U5 snRNP components *EFTUD2* and *DDX23*. This suggests an increased splicing regulatory activity along the tumor progression trajectory.

We next performed differential alternative splicing analysis, identifying two major AS event clusters based on hierarchical clustering (Fig. S6C–E). Cluster 1, which was upregulated in State 2 (differentiated normal epithelium), was enriched for retained intron (RI) events, including *EWSR1*, *CDH17*, and *CD151*. This suggests downregulation of intron retention in State 3 (cancer cells), indicating a distinct AS regulatory mechanism associated with tumor progression.

Together, these findings uncover a dynamically regulated splicing landscape along the CRC differentiation trajectory, emphasizing the potential functional significance of splicing regulation in tumor progression.

### Isoform diversity and alternative splicing in iCMS subtypes

Colorectal cancers have been classified into intrinsic-consensus molecular subtypes (iCMS) based on single-cell transcriptome profiles (Joanito et al., 2022). iCMS2 is characterized by elevated MYC and WNT pathway activation and higher levels of copy number variations (CNVs), whereas iCMS3 exhibits increased MAPK pathway activity and fewer copy number alterations. To gain deeper insights into the molecular distinctions between these subtypes, we further investigated isoform diversity within the iCMS subtypes.

We first computed module scores for iCMS2 and iCMS3 signatures in individual cells using previously identified marker genes for these subtypes (Joanito et al., 2022). Twelve CRC patients were assigned into iCMS2 (CRC01, CRC04, CRC05, CRC08, CRC09, CRC10, HTCRC01, HTCRC05) and iCMS3 (CRC02, CRC03, CRC07, HTCRC04) type (Fig. S7A). Both scRNA-seq-inferred CNV profiles and WES CNV data revealed that iCMS2 tumors harbored more somatic CNAs than iCMS3 tumors (Figs. 1F and S7B). Moreover, gene set enrichment analysis (GSEA) comparing iCMS2 and iCMS3 in our dataset aligned with previous findings (Joanito et al., 2022), demonstrating that MYC activity was mainly upregulated in iCMS2 cancer cells, whereas epithelial-mesenchymal transition (EMT) and TGF-beta signaling were enriched in iCMS3 cancer cells (Fig. S7C). These results are consistent with prior reports and further validate the accuracy of our iCMS patient classification.

**Figure 5.**
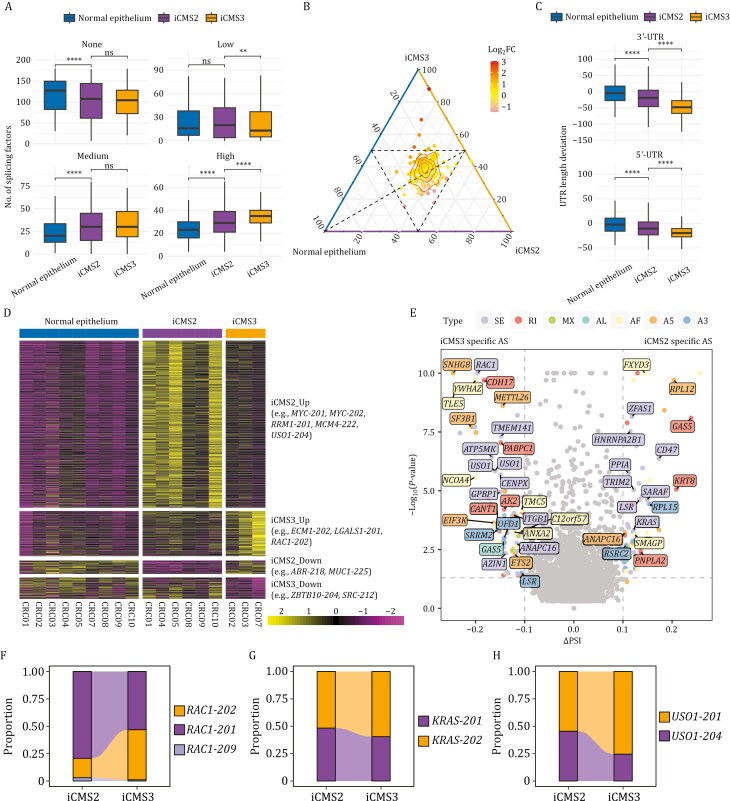
Transcript level characteristics in iCMS2 and iCMS3 epithelial long-read transcriptomes. (A) Box plot showing the number of splicing factors grouped by low, medium, and high expression levels in normal epithelial cells, iCMS2 and iCMS3 cancer cells. Statistical significance was determined using the two-tailed Wilcoxon rank-sum test, with ns indicating no significant difference, **P < 0.01, and ****P < 0.0001. (B) Ternary plot depicting the expression distribution among normal epithelial cells, iCMS2, and iCMS3 cancer cells. Each point represents a splicing factor, with its position indicating the relative expression across the three groups. Color intensity represents the log₂(fold change) (log_2_FC) between iCMS3 versus iCMS2, with red indicating upregulation and purple indicating downregulation. Contour lines denote the density of genes within specific regions. The dashed triangle highlights the central cluster of gene expression patterns. (C) Box plot showing the length deviation of 3′-UTR (top) and 5′-UTR (bottom) in normal epithelial cells, iCMS2 and iCMS3 cancer cells. Statistical significance was determined using the two-tailed Wilcoxon rank-sum test, with ****P < 0.0001. (D) Heatmap showing differentially expressed transcripts (DETs) across iCMS2, iCMS3, and normal epithelium samples. Significant differential expressed transcripts were classified as “Up” or “Down” in an iCMS subtype only if they were consistently upregulated or downregulated compared to both the other iCMS subtype and normal epithelium, with a fold change ≥ 2 and a *P*-value < 0.05. (E) Volcano plot displaying significant differentially alternative splicing (DAS) events between iCMS2 and iCMS3 subtypes. Significant differential alternative splicing events were defined as iCMS2 specific or iCMS3 specific only if it was consistently upregulated relative to both of the other iCMS subtype and normal epithelium, with ΔPSI ≥ 0.1 and *P*-value < 0.05. Each labeled point represents an alternatively spliced gene. SE, skipping exon, RI, retained intron, A5, alternative 5′ splice-site, A3, alternative 3′ splice-site, AF, alternative first exon, AL, alternative last exon, MX, mutually exclusive exons. (F–H) Stacked bar plot showing isoform proportions of *RAC1* (F), *KRAS* (G) and *USO1* (H) across iCMS2 and iCMS3 subtypes.

We also found that both iCMS2 and iCMS3 subtypes exhibited greater splicing factor expression compared to normal epithelial cells. Furthermore, for these splicing factors, iCMS3 showed slightly higher activities than iCMS2 (Fig. 5A and 5B). In parallel, we observed a consistent trend of UTR shortening in both iCMS2 and iCMS3 subtypes relative to normal epithelial cells, with iCMS3 displaying the most pronounced shortening (Fig. 5C). This pattern suggests distinct post-transcriptional regulatory alterations, potentially affecting mRNA stability, translational efficiency, and gene expression dynamics in a subtype-specific manner.

Next, we performed differential transcript expression analysis on epithelial cell long-read transcriptomes. RNA isoforms upregulated in iCMS2 include *MYC-201*, *MYC-202*, *RRM1-201,* and *MCM4-222* in the *MYC* target gene sets, while RNA isoforms upregulated in iCMS3 include *ECM1-202* and *LGALS1-201* in the epithelial-mesenchymal transition dataset (Fig. 5D). We further investigated alternative splicing patterns to characterize the transcriptomic differences between iCMS2 and iCMS3. A total of 46 significantly differential alternative splicing events were identified (Fig. 5E; Table S6). For example, among isoforms involved in exon skipping of *RAC1* (SE:7:6392041-6398662:6398718-6400126:+), the proportion of *RAC1-202* (corresponding to Rac1b) was significantly upregulated, while *RAC1-201* (corresponding to Rac1) was significantly downregulated in iCMS3 (Fig. 5F). This finding is consistent with a previous study showing that Rac1b promotes tumor cell migration (Fu, 2017), characteristic of iCMS3 tumors. Similarly, we also identified differences in isoform usage between *KRAS-201* (KRAS4a) and *KRAS-202* (KRAS4b), as well as between *USO1-201* and *USO1-204* (Fig. 5G and 5H), which have been reported as functionally relevant in cancer (Chen et al., 2021b; Dong et al., 2022).

These findings indicate extensive isoform diversity and alternative splicing in colorectal cancer subtypes iCMS2 and iCMS3, with subtype-specific splicing events potentially contributing to the underlying molecular heterogeneities.

### Differential transcript usage in colorectal cancer

**Figure 6.**
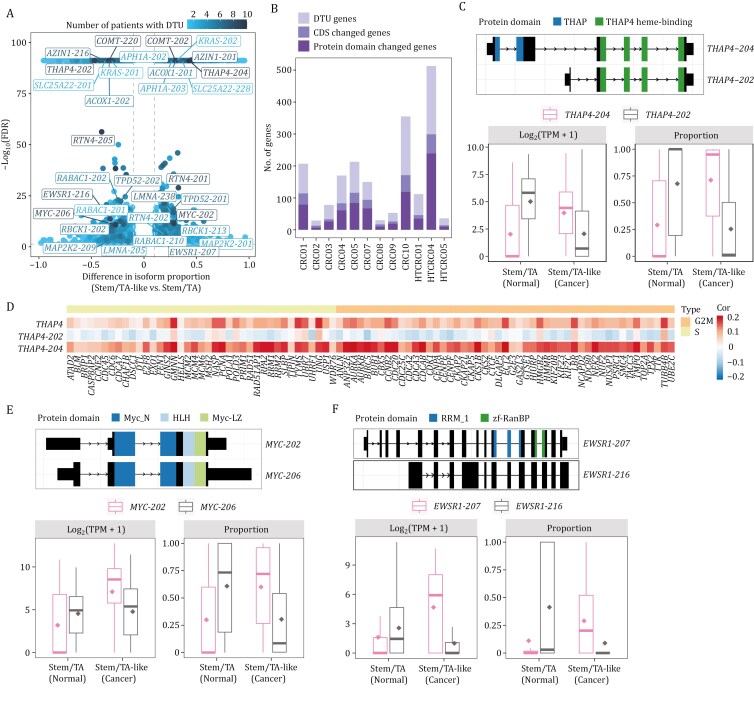
Analysis of differential transcript usage in CRC. (A) Volcano plot showing differential transcript usage between stem/TA-like cells and stem/TA cells in CRC. Each dot represents a transcript isoform, with color indicating the number of patients in which the DTU is observed. The x-axis indicating the average proportion difference of DTU genes across corresponding patients, and the y-axis showing the minimum statistical significance (− log_10_FDR) among those patients. (B) Bar plot showing the number of genes exhibiting CDS changes among those exhibiting DTU, and the number of genes with protein domain alterations among those with CDS changes. (C) Schematic of THAP4 protein domains (top) and box plots showing the absolute expression (log_2_(TPM + 1)) and relative proportion of *THAP4* isoforms in stem/TA and stem/TA-like cells (bottom). (D) Heatmap with hierarchical clustering showing the correlation between absolute expression of the *THAP4* gene and its isoforms with cell cycle-related genes across different phases. (E) Schematic of MYC protein domains (top) and box plots showing the absolute expression (log_2_(TPM + 1)) and relative proportion of *MYC* isoforms in stem/TA and stem/TA-like cells (bottom). (F) Schematic of EWSR1 protein domains (top) and box plots showing the absolute expression (log_2_(TPM + 1)) and relative proportion of *EWSR1* isoforms in stem/TA and stem/TA-like cells (bottom).

To explore the contribution of transcript composition differences at the gene level, we performed differential transcript/isoform usage (DTU or DIU) analysis (Methods). We identified 3,548 unique transcripts from 1,946 genes that exhibited significant proportional differences between stem/TA-like cells (cancer cells) and stem/TA cells (normal epithelial cells) across 12 CRC patients (Fig. S8A; Table S7). Top genes identified with significant DTU included *THAP4*, *RTN4*, *ACOX1*, *MYC*, *LMNA*, *EWSR1*, *RBCK1*, *TPD52*, *AZIN1*, *COMT*, *RABAC1*, *APH1A*, *MAP2K2*, *KRAS,* and among others (Fig. 6A). The oncogenes *MAP2K2* and *KRAS* exhibited changes in isoform proportions, despite no significant alterations in their overall gene expression levels, which indicates that their distinct RNA isoforms may have different functions in colorectal cancer. In addition, the proportion of the *APH1A-203* and *RABAC1-201* isoforms, which were upregulated in our data, exhibited a progressive increase across cancer stages in the TCGA cohort. Conversely, the proportion of the downregulated *APH1A-202* and *RABAC1-202* isoforms displayed a decreasing trend with disease advancement (Fig. S8B–E). Further, we identified 63 RNA isoforms whose altered proportions correlated with the survival outcomes of the patients. Notably, if counted at the individual gene level instead of individual RNA isoform level, 60 of these isoforms showed no correlation with the survival of the patients, highlighting the importance of isoform-level analysis. Isoforms with increased proportions in stem/TA-like cells (cancer cells) were linked to worse survival, such as *COMT-202*, *TTC39A-216*; while increased proportions in stem/TA-like cells (cancer) indicated better prognosis, such as *STAG2-203* and *CCND3-203* (Fig. S8F). These findings suggest a potential clinical relevance of these isoform-specific expression dynamics, highlighting their possible roles in tumor progression and molecular subtyping.

Interestingly, on average, 74.0% of the DTU genes in each patient did not overlap with the differentially expressed genes, suggesting the masked heterogeneities that often exists within a single gene locus (Fig. S9A). There is also minimal overlap between DTU transcripts and differentially expressed transcripts, suggesting that changes in absolute abundance do not necessarily reflect alterations in isoform usage, which may indicate preferential transcript selection by genes under specific conditions (Fig. S9B). Notably, an average of 50.0% of DTU genes exhibited significant coding sequence (CDS) alterations exceeding 20 bp, with a median CDS length difference of 445 bp and changes of three exons. Furthermore, 70.1% of these genes underwent isoform switches that led to changes in protein domains, affecting an average of two domains per DTU gene (Figs. 6B, S9C–E and Methods).

For example, *THAP4* exhibited a shift in transcript dominance from *THAP4-202* (which does not contain the THAP domain) in stem/TA cells (normal epithelial cells) to *THAP4-204* (which contain the THAP domain) in stem/TA-like cells (cancer cells) (Fig. 6C). This suggests that the THAP domain of *THAP-204*, which is a conserved zinc-finger motif associated with transcriptional regulation, cell cycle control, and apoptosis (Bessiere et al., 2008; Roussigne et al., 2003), appears to have a more pronounced oncogenic role when upregulated compared to the THAP4 heme-binding domain. Furthermore, the expression of *THAP-204* is positively correlated with that of cell cycle-related genes, while *THAP-202* is negatively correlated, suggesting their opposing roles in the regulation of cell cycle (Fig. 6D). The well-known proto-oncogene c-Myc encodes distinct proteins, including c-Myc1 (*MYC-206*) and c-Myc2 (*MYC-202*), which are initiated from CUG and AUG codons respectively. In stem/TA-like cells (cancer cells), we observed a shift in isoform usage, with reduced *MYC-206* and increased *MYC-202* deployment (Fig. 6E). This suggests distinct regulatory roles for *MYC-202* and *MYC-206* in carcinogenesis, consistent with studies indicating that c-Myc2 predominantly drives cell growth and proliferation, while c-Myc1 inhibits growth in the absence of c-Myc2 (Hann et al., 1994; Liao and Dickson, 2000). Another instance of altered isoform usage is the shift between *EWSR1-207* and *EWSR1-216*, with the latter retaining an intron that prevents protein production. In stem/TA-like cells (cancer cells), there was a clear preference for *EWSR1-207*, which encodes a protein critical for preventing hematopoietic stem cell senescence (Lee et al., 2019), rather than *EWSR1-216* (Fig. 6F). Similarly, we observed consistent isoform usage patterns in four colorectal cancer cell lines, further supporting our *in vivo* findings (Fig. S9F–H).

To further explore isoform diversity at the level of functional coding units, we conducted a differential CDS usage (DCU) analysis, focusing specifically on the protein-coding potential of the transcriptome rather than its compositional variations. This analysis identified a total of 773 unique CDS regions across 381 genes that exhibited significant proportional differences between stem/TA-like cells (cancer cells) and stem/TA cells (normal epithelial cells) from 12 CRC patients (Fig. S10A; Table S8 and Methods). On average, 85.2% of the DCU genes per patient overlapped with CDS-changed DTU genes, suggesting the notion that DTU may serve as an indicator of potential protein expression differences (generating different proteins) from the same gene (Fig. S10B and S10C).

In short, isoform shifts in colorectal cancer appears to be a significant post-transcriptional regulatory mechanism, systematically influencing protein sequences and function and contributing to cellular ecosystems that underpin therapeutic responses and resistance, suggesting potential targets for intervention.

### Allele-specific expression revealing somatic mutational signatures of transcriptome in colorectal cancer

**Figure 7.**
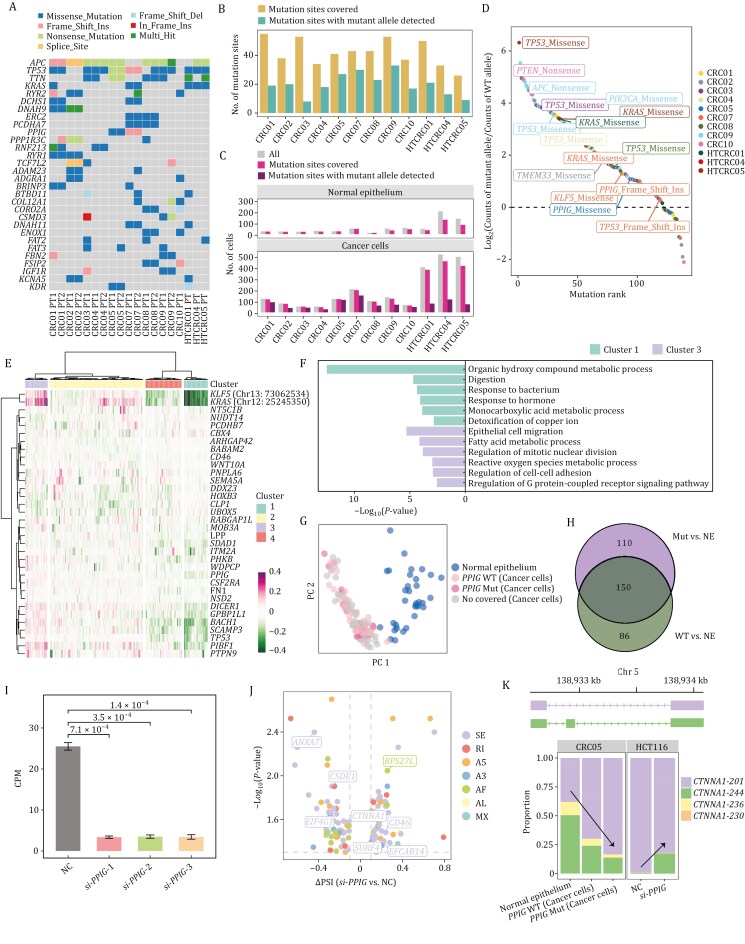
Transcriptional mutation signatures at single-cell resolution in CRC. (A) Oncoplot showing the top 30 recurrently altered genes of 19 tumor-normal pairs from the 12 patients. (B) Bar plot showing the number of genes with mutation sites covered (yellow) and mutation sites with mutant allele detected (green). (C) Bar plots showing the number of cells in normal epithelium and cancer cells in each patient. Gray represents all cells sequenced in this profiling. Pink represents that cell with at least one mutation site covered. Purple represents the cells with at least one mutation site with mutant allele detected. (D) Dot plot showing coverage ratio of mutant alleles to wild-type alleles at each mutation site in mutated cancer cells. (E) Heatmap of the correlation difference between $R_{mut\cdot hvg}$ and $R_{wt\cdot hvg}$ in patient CRC07, which $R_{mut\cdot hvg}$ represents Pearson correlation coefficients between mutant allele expression and highly variable gene expression, and $R_{wt\cdot hvg}$ represents Pearson correlation coefficients between WT allele expression and highly variable gene expression. (F) Bar plot showing the GO terms enriched for genes of which the expression level negatively (green) and positively (purple) associated with that of the mutant allele, respectively. (G) PCA plot of cells in patient CRC05, colored by the presence or absence of *PPIG* mutations. (H) Venn diagram showing the number of differential alternative splicing events between *PPIG* WT cells versus normal epithelial cells (green) and *PPIG* mutant cells versus normal epithelial cells (purple). (I) Gene expression level of *PPIG* following siRNA-mediated knockdown in HCT116 cells, quantified as counts per million (CPM). Bars represent the mean CPM values for *PPIG* across three independent siRNAs targeting *PPIG* (si-*PPIG*-1, si-*PPIG*-2, si-*PPIG*-3; each with 3 replicate samples) and the corresponding negative control (NC, 3 replicate samples). Data are shown as mean ± SEM, with *P*-values indicating statistical significance from pairwise comparisons using Student’s *t*-test. (J) Volcano plot depicting the significance and magnitude of differential AS events between *PPIG* knockdown and NC groups. Each dot represents a splicing event, with the x-axis indicating difference in percent spliced in (ΔPSI) and the y-axis representing the –log_10_(*P*-value). Colors correspond to the AS types. (K) Proportions of transcript isoforms involved in the overlapping AS events for *CTNNA1* in CRC05 and HCT116 cells. Arrows indicate the direction of PSI changes.

Given the full-transcript coverage, long-read scRNA-seq enables a broader exploration of transcriptional mutation signatures at single-cell resolution. To define a spectrum of high-confidence somatic mutations in the expressed genome as reference, the gold standard whole-exome sequencing (WES) was conducted on 31 tissue samples from the 12 patients (Methods). A total of 1,228 unique somatic alterations from 1,110 genes were identified in the primary tumors with matched normal tissue as control within each patient (Fig. 7A; Table S9).

Subsequently, we focused on allelic expression and performed a pileup of genome mapping reads at mutation sites in the long-read scRNA-seq data to determine the coverage of reference/wild-type (WT) and alternative/mutant alleles in individual cells, respectively, generating three allelic expression matrices: WT allele × cell expression matrix ($E_{wt}$); mutant allele × cell expression matrix ($E_{mut}$); total × cell expression matrix ($E_{tot}$) (Methods). 473 expressed genes were covered within mutation sites, and 46% (219 genes) showed the presence of mutant alleles using the exact binomial test (Fig. 7B). Cancer cells exhibited prevalent mutations, while normal cells exhibited essentially no detectable mutations, confirming that the observed mutations are tumor-specific (Figs. 7C, S11A and S11B).

Furthermore, we found that cancer cells exhibited dominant expression of the mutated alleles (Fig. 7D), including those in the proto-oncogene *KRAS* (e.g., c.35G>T in CRC07, c.436G>A in HTCRC01 and c.38G>A in HTCRC05), *KLF5* (c.935C>T in CRC07) and *PIK3CA* (c.2836G>T in CRC09). Interestingly, dominant mutant allele expression was also identified in tumor suppressor genes, including *APC* (c.3871C>T, resulting in a truncated version of APC protein (Kim et al., 2005; Moseley et al., 2007) in CRC09), *PTEN* (c.697C>T, resulting in a truncated version of PTEN protein (Bonneau and Longy, 2000; Lee et al., 2010) in CRC10) and *TP53* (c.473G>A (Pros et al., 2020) in CRC03, c.455C>T (Masciari et al., 2011; Yurgelun et al., 2017) in CRC04, c.818G>A (Petitjean et al., 2007; Chang et al., 2018) in CRC09 and c.524G>A in HTCRC05), which may have oncogenic implications in specific contexts. Distinct from prior research of short-read scRNA-seq technology, we simultaneously achieved the mutations’ genotype and expression phenotype at single-cell resolution in colorectal cancer.

To disentangle the transcriptional phenotypic effect of these mutations among individual cancer cells, we calculated Pearson correlation coefficients between allelic expression matrices of mutation sites ($E_{tot}$, $E_{wt}$ and $E_{mut}$) and gene expression matrix of highly variable genes ($E_{hvg}$) within each patient to produce correlation matrices $R_{tot\cdot hvg}$, $R_{wt\cdot hvg}$ and $R_{mut\cdot hvg}$, respectively (Fig. S11C and Methods). We noticed a stark shift in correlation pattern in patient CRC07 between WT and mutant alleles of *KRAS* and *KLF5*, as is evident in the differential correlation matrix ($R_{mut\cdot hvg}-R_{wt\cdot hvg}$, Fig. 7E). In particular, their mutant alleles showed negative correlation with genes in cluster 1 and positive correlation with genes in cluster 3, whereas the WT alleles showed a reversed trend. The mutant-associated genes in cluster 3 were dominantly related to pro-cancer biological processes including cell migration, mitotic nuclear division and cell-cell adhesion, in contrast to the WT-associated genes in cluster 1 enriched in physiological functions of the intestinal tract (Fig. 7F). This suggests that mutations in *KRAS* and *KLF5* may be associated with specific adaptive transcriptional programs to tumorigenesis in CRC through distinct correlations with these highly variable genes. These results illustrate the concealed functional impact of mutations on the cells’ transcriptional phenotype, which can be masked when only inspecting total expression level of each gene. Similar correlation shift caused by somatic mutations were also observed in other patients but with different genes, reflecting the intra-tumor and inter-tumor heterogeneities in transcriptome plasticity of colorectal cancer (Fig. S11D).

We next surveyed mutated splicing factor genes and analyzed their effects on RNA splicing process. We found that a splicing factor *PPIG* was mutated in patient CRC05, which has also been previously identified in multiple cancer types (Seiler et al., 2018). Differential alternative splicing analysis was conducted for *PPIG* mutant and *PPIG* wild-type cancer cells, respectively, in comparison with normal epithelium (Figs. 7G, 7H, S12A and S12B). Splicing alterated genes in *PPIG* WT cancer cells were linked to cellular stability, mitochondrial integrity, and stem cell maintenance, indicating a more balanced regulation of survival and differentiation. In contrast, splicing alterated genes in *PPIG* mutant cancer cells were associated with cell cycle regulation and cellular organization processes, potentially promoting more aggressive tumor behavior (Fig. S12C). This result indicated that this *PPIG* mutation is a gain-of-function one by altering RNA splicing to promote oncogenic processes.

To further investigate the role of *PPIG* in modulating RNA splicing in colorectal cancer, we knocked down its expression in human colorectal carcinoma cell line HCT116 using small interfering RNA (siRNA) transfection and performed long-read RNA sequencing. As expected, *PPIG* knockdown using three different siRNAs all resulted in a significant reduction in its transcript abundance, confirming the efficiency of the perturbation (Fig. 7I). To investigate the role of *PPIG* in modulating RNA splicing, we performed differential alternative splicing analysis between *PPIG* knockdown and negative control (NC) groups. We first examined the consistency between *in vivo* and *in vitro* data and found that 45 out of 69 AS events exhibited consistent changes across both conditions. Since the *PPIG* mutation we identified *in vivo* is expected to be a gain-of-function mutation, the knockdown of *PPIG* in the HCT116 cell line *in vitro* should lead to the reverse of the downstream effects of *PPIG* mutation in the cancer cells of Patient CRC05 *in vivo* (Here, we only considered AS events with a PSI difference greater than 5%, without requiring statistical significance). A total of 133 DAS events were identified, with exon skipping being the most prevalent event type. Among these, 106 DAS events resulted in coding sequence changes, which could disrupt the protein sequences of the corresponding genes (Figs. 7J and S12). Notably, eight differential alternative splicing events significantly overlapped between *PPIG* knockdown vs. NC groups in HCT116 and *PPIG* mutant cancer cells vs. normal epithelial cells in CRC05 (hypergeometric test, *P*-value = 0.015, Fig. 7J). The proportions of transcripts involved in these AS events of genes were rescued after *PPIG* knockdown (Figs. 7K and S12E), such as *CTNNA1* (catenin alpha 1, a component of the cell adhesion complex), *EIF4G1* (eukaryotic translation initiation factor 4 gamma 1, essential for translation initiation), *CD46* (complement regulatory protein involved in immune response modulation), *CSDE1* (cold shock domain-containing E1, a regulator of RNA stability and translation), and *SURF4* (surfeit locus protein 4, associated with protein trafficking and secretion).

In addition, GO enrichment analysis revealed that these 133 DAS genes are primarily involved in translation, DNA repair, cell cycle regulation, RNA stabilization, and mitochondrial transport (Fig. S12F), which aligns with the biological processes observed in CRC05 cancer cells compared with normal epithelial cells. Furthermore, GSEA between *PPIG* knockdown vs. NC groups revealed that *PPIG* knockdown could reduce the activities of some oncogenic pathways, as shown in enrichment plots for gene sets EGFR_UP.V1_UP, KRAS.DF.V1_UP, and MEK_UP.V1_UP (Fig. S12G).

Collectively, these results demonstrate the consistency of *in vitro* validation data with *in vivo* data from patient CRC05. *PPIG* is shown to play a crucial role in maintaining RNA splicing fidelity and regulating cancer-associated splicing programs. These findings suggest that targeting *PPIG* may serve as a potential therapeutic strategy for colorectal cancer by simultaneously correcting splicing defects and suppressing tumor-promoting signaling pathways.

## Discussion

This study delves into the intricate heterogeneities of colorectal cancer at both cellular and molecular dimensions, with a particular focus on the complexities of RNA isoforms. We performed long-read scRNA-seq on the epithelium of CRC tumor and normal samples from 12 patients to define the full-length isoform-level transcriptome. We achieved a high-fidelity, premium-quality dataset with each individual cell, underpinning a robust basis for quantitative abundance-based analyses. Through isoform-level analysis, our study delineated diverse cell subtypes, with validation against a large external dataset confirming the reliability of cell type identification, and revealed a rich tapestry of isoform diversities within specific gene markers. Additionally, we found a significant number of isoforms to be differentially regulated in cancer cells, with upregulation of splicing-related processes.

Following this, our analysis utilizing SQANTI3 to compare splice junctions against reference transcripts facilitated the categorization of the 27,140 isoforms into four categories: FSM, ISM, NIC and NNC, with the majority being FSM isoforms. Cancer cells exhibited a higher number and variety of isoforms and genes than normal epithelial cells, a trend that persisted even after normalizing for both cell numbers and sequencing depths. Despite stringent filtering, the proportion of novel transcripts was slightly higher in cancer cells, suggesting a higher rate of alternative splicing events, contributing to greater transcriptomic diversities in CRC. This was further supported by the observation that half of the genes in cancer cells expressed multiple isoforms, compared to 43% in normal epithelium.

By establishing gene UTR deviation as a measure of UTR length variations in individual cells relative to the baseline of normal epithelium, we uncovered a pervasive trend of 3′-UTR shortening within cancer cell populations, particularly among stem/TA-like cells. And we identified a set of genes with shortened and lengthened 3′-UTRs. Notably, shortened 3′-UTRs were prevalent in genes associated with RNA processing and metabolic processes and were often correlated with increased RNA expression levels, implying a potential mechanism of shortened 3′-UTRs for increased mRNA stability and translational efficiency as well as altered gene regulation through avoidance of microRNA binding. However, lengthened 3′-UTRs did not exhibit a consistent pattern of correlation with RNA levels. These insights collectively highlight 3′-UTR shortening as a distinctive hallmark of colorectal cancer, potentially playing a critical role in the aberrant gene expression landscape characteristic of the disease.

Our findings revealed extensive alternative splicing alterations in colorectal cancer, with reduced intron retention in stem/TA-like cancer cells and significant inter-patient heterogeneities. Correlation analysis identified key splicing factors, including *HSPA8*, *SNRPB*, and *hnRNPs*, linked to tumor-specific AS events, especially for reduced intron retention. Pseudotime analysis further showed increased SF expression and splicing activity during tumor progression. In addition, we revealed extensive RNA isoform diversities and alternative splicing differences between iCMS2 and iCMS3 colorectal cancer subtypes. iCMS3 exhibited even higher splicing factor activity and more pronounced 3′-UTR shortening than iCMS2, suggesting distinct post-transcriptional regulation. Differential isoform usage, including RAC1b upregulation in iCMS3, aligns with its aggressive phenotype.

Notably, we discovered significant isoform switches that may alter protein function from the same gene, suggesting new avenues for targeted cancer therapy. Many DTU genes, including oncogenic genes like *KRAS*, *MYC*, and *EWSR1*, exhibited isoform shifts independent of overall gene expression, emphasizing functional diversification. Notably, these isoform switches frequently altered coding sequences and protein domains from the same gene, potentially influencing cancer progression. The correlation between DTU events and patient survival further underscores their clinical relevance.

Leveraging the comprehensive transcriptome coverage offered by long-read scRNA-seq, our study has pushed the boundaries of transcriptional mutation profiling at the single-cell level. We demonstrate that cancer cells exhibit dominant expression of mutated alleles in key oncogenes and tumor suppressor genes, probably through gene locus amplification or enhanced transcriptional activities of the mutant allele. Mutant allele-specific transcriptional shifts were linked to oncogenic processes such as cell migration and mitotic division, highlighting their functional relevance during tumorigenesis. Additionally, mutations in the splicing factor *PPIG* led to widespread alternative splicing changes, which were validated through perturbation experiments in colorectal cancer cells.

Taken together, our employment of long-read scRNA-seq has uncovered significant isoform heterogeneities and distinct splicing regulation in colorectal cancer. We emphasized the prevalence of 3′-UTR shortening, the impactful role of somatic mutations on transcriptomic state of cancer cells, and the alterations in protein function due to isoform switching on the transcriptomic landscape, indicating potential new targets for therapy. Further studies should expand the scope to include a larger cohort of patients, diverse cancer types, and further exploration of the therapeutic implications of splicing-related mutations could provide deeper insights into the mechanistic roles of transcriptomic alterations in cancer progression and treatment resistance.

## Methods

### Sample collection

We collected samples from 12 patients who were clinically diagnosed with microsatellite-stable CRC, and none of them was metastatic. Primary tumors and matched adjacent normal tissues were obtained from all 12 patients.

### Processing of human tumor samples

Dissected tumor samples and matched normal mucosa were digested into single-cell suspension via 1.5 mg/mL collagenase type II (Gibco, 17101015) and 1.5 mg/mL collagenase type IV (Gibco, 17104019). All samples were treated at 37°C for 30–60 min, depending on viscosity. We used CD45 antibody (BioLegend, 368510) and EPCAM antibody (BioLegend, 369810) in FACS to collect CD45^−^EPCAM^+^ epithelial cells. Cells were sorted into 96-well plates containing SCAN-seq lysis buffer and stored at −80°C.

### SCAN-Seq single-cell cDNA amplification

Plates were thawed and thoroughly vortexed for 60 s, and incubated at 72˚C for 3 min to release the linearized RNA molecules and were immediately transferred on ice. Reverse transcription and PCR amplification were performed as described before. In brief, for SCAN-seq (Fan et al., 2020), each well was added 2.85 μL RT mixture (50 U SuperScript II reverse transcriptase, 5 U RNAse inhibitor, 5× Superscript II first-strand buffer, 25 mmol/L DTT, 5 mol/L Betaine, 30 mmol/L MgCl_2_ and 5 μmol/L TSO). We ran the RT reaction at 25°C for 5 min, 42°C for 60 min, 50°C for 30 min, and 70°C for 10 min. Then, a 7.5-μL PCR mixture containing 2× KAPA HiFi HotStart ReadyMix and 200 nmol/L IS PCR primers was added. The PCR reaction was performed following the program: 95°C for 3 min, 4 cycles at 98°C for 20 s, 65°C for 30 s, and 72°C for 5 min, followed by 20 cycles at 98°C for 20 s, 67°C for 15 s, and 72°C for 5 min, with a final cycle at 72°C for 5 min. For SCAN-seq2 part (Liao et al., 2023), each well was added 2.85 μL RT mixture (1 mol/L Tris-HCl pH 8.3, 1 mol/L NaCl, 1 mol/L MgCl_2_, 5 mmol/L GTP, 40 mmol/L DTT, 10 μmol/L TSO, 10 U Maxima H-minus RT enzyme, RRI and 50% PEG4000). RT reaction was performed at 42°C for 90 min, 11 cycles for 50°C for 2 min, 42°C for 2 min, and 85°C for 15 min. Plates were spun down and transferred to ice immediately. Every 32 cells with different 3′ barcodes were pooled together and purified with 0.8× Ampure XP beads. After that, the PCR mixture including 2× KAPA HiFi Hot-Start Ready Mix, 266 nmol/L 3′ P2 primer, and 266 nmol/L IS PCR primer was added into each tube. The amplification was performed by the following program: 95°C for 3 min, 4 cycles at 98°C for 20 s, 62°C for 30 s, and 72°C for 5 min, followed by 23 cycles at 98°C for 20 s, 67°C for 15 s, and 72°C for 5 min, with a final cycle at 72°C for 5 min. We pooled the cDNAs of different cell barcodes together and purified them twice with Ampure XP beads (Beckman, Brea, CA, USA, Cat# A63882). A total of 500 ng to 1 μg cDNA products were used for further library construction.

### SCAN-seq library preparation and sequencing

Ligation Sequencing Kit 1D (SQK-LSK110, Oxford Nanopore Technologies) was used to prepare full-length cDNA libraries with the following instructions: cDNA fragments were end-repaired and dA-tailed using NEBNext Ultra II End Module, then tethered to 1D adapter by using Quick Ligation Module. Each cDNA library was loaded into one PromethION FLO-PRO002 flow cell and sequenced on PromethION.Beta.

### Exome library preparation and construction

Genomic DNAs were extracted from tumor samples and normal mucosa, and then fragmented into 150–200 bp. After end repair and adaptor ligation, DNA was amplified with NEB primers and 2× KAPA HiFi HotStart ReadyMix (Kapa Biosystems, Cat# KK8054). Libraries with different barcode adaptors were finally pooled together for whole-exome sequence.

### Cell culture of colorectal cancer cell lines

The human colorectal carcinoma cell line 116 (HCT116) was grown in RPMI 1640 medium supplemented with 10% fetal bovine serum (FBS), 1% penicillin-streptomycin. LoVo human colon adenocarcinoma cell line (LoVo) was cultured in DMEM medium supplemented with 10% fetal bovine serum (FBS), 1% penicillin-streptomycin. The human colon adenocarcinoma cell line 29 (HT29) and the southwest colon adenocarcinoma cell line 480 (SW480) were cultured in DMEM medium, supplemented with 10% fetal bovine serum (FBS), 1% penicillin-streptomycin, and 1% L-glutamine.

### SCAN-Seq bulk cell line sequencing

For each cell line, 500,000 cells were collected and total RNA was extracted by RNase Mini kit (QIAGEN, 74106). Samples met the quantity standard (RIN higher than 7, overall amount higher than 0.9 μg, concentration higher than 30 ng/μL) and purity standard (OD_260_/OD_280_: 1.8–2.2, OD_260_/OD_230_: 1.3–2.5, Nc/Qc lower than 2.5) were further used to library construction and sequencing.

### SCAN-seq data preprocessing and transcript assembly

The electronic raw signals (FAST5 data) were translated into bases (FASTQ data) using Guppy (v3.1.5). Nanoplexer (v0.1) was performed on the FASTQ data to generate individual cell data of SCAN-seq based on the 24 bp barcode information. Reads with barcode mapping score below 31 were discarded (nanoplexer -s 31). Low-quality reads (read quality score < 7) and short reads (read length < 100) were then filtered using NanoFilt (v2.8.0) (De Coster et al., 2018). In order to obtain full-length reads, PCR anchor sequence and cell barcode sequence were identified and trimmed using Pychopper (v2.7.2), and poly(A) tail was trimmed using trim_isoseq_polyA command. The full-length reads were aligned to the human reference genome (GRCh38, Ensembl, release-108) using minimap2 (v2.17) (-ax splice -uf -k14 --secondary = no --junc-bed) (Li, 2018). All bam files were consolidated into a single bam file, followed by transcriptome assembly conducted by StringTie (Kovaka et al., 2019). Transcript isoforms with coverage below 3 were subsequently eliminated.

### Identification and quantification of assembled transcripts

The assembled full-length transcriptome was quality-controlled and filtered using SQANTI3 (Tardaguila et al., 2018) (v5.1), respectively, with the aid of the sqanti3_qc.py and sqanti3_filter.py scripts. Based on the comparison between the reference transcriptome (Ensembl, release-108) and transcript splice junctions, SQANTI3 classify isoforms into 7 classes: Full Splice Match (FSM), Incomplete Splice Match (ISM), Novel in Catalog (NIC), Novel Not in Catalog (NNC), Intergenic, Genic Intron and Genic Genomic. Only known transcripts of known genes (FSM and ISM) and novel transcripts of known genes (NIC and NNC) were considered in this project. The coordinates of FSM and ISM isoform were revised to be consistent with the annotation of the reference transcriptome for more accurate quantification in subsequent analyses. The counts of transcript isoforms and genes of each single cell were quantified using StringTie (v2.1.7) using assembled transcriptome as reference. Transcript isoforms were further filtered according to the following standards: (i) the isoform was expressed in at least three cells per individual; (ii) expressed in at least three individuals. Overall, a corrected transcript annotation set of 27,140 nonredundant isoforms from nine colorectal cancer patients was established.

The expression levels were calculated as read counts per 1,000,000 mapped full-length reads for each gene and transcript (TPM) and transformed by log(TPM/10 + 1), which presents expression level unless otherwise mentioned. Cells with less than 500 genes detected and more than 60% mitochondrial counts were discarded.

### Dimension reduction and cell-type identification

The R package Seurat (v4.3.0) (Hao et al., 2021) was conducted to perform dimension reduction and clustering analysis using the transcript count matrix. A total of 3,262 cells from SCAN-seq (9 CRC patients) and SCAN-seq2 (3 CRC patients) datasets were used to create a single Seurat object. Top 1000 highly variable genes (HVGs) were selected respectively from SCAN-seq and SCAN-seq2 data. The union of HVGs were used to perform principal component analysis (PCA) and top 20 significant principal components (PCs) were selected to construct a K-nearest neighbor (KNN) graph, followed by clustering using the Louvain algorithm with a Shared Nearest Neighbor (SNN) modularity optimization. t-SNE was applied to visualize clusters and cell type distributions. Based on the expression of cell type-specific marker genes and sample collection information, malignant cancer cells and nonmalignant normal epithelial cells were identified.

For normal epithelial cells, a similar process was applied for further dimension reduction and cell clustering using the union of top 1000 HVGs from SCAN-seq and SCAN-seq2 datasets, with the top 30 significant PCs and an RNA_snn resolution of 2.5, resulting in the identification of these 13 distinct clusters. Normal epithelial cells were categorized into five cell types based on marker gene expression: stem/TA cells, enterocytes, immature goblet cells, goblet cells and enteroendocrine cells. Cancer cells were further classified into three cell types: stem/TA-like cells, immature goblet-like cells and goblet/enterocyte-like cells using reference component analysis (RCA) (Li et al., 2017), with the normal epithelial cell types serving as reference.

We extracted approximately 168,000 epithelial cells from the Human Colon Cancer Atlas dataset (c295, GSE178341) (Pelka et al., 2021) and randomly divide these cells into 5 equal-sized cohorts followed by integration with our data respectively. Each integration was conducted by canonical correlation analysis (CCA) as implemented in Seurat package, using the top 1000 highly variable genes and the top 30 significant PCs.

### Differential gene expression analysis and differential transcript expression analysis

To identify differential gene expression (DGE) and differential transcript expression (DTE) genes between specific two cell types, we utilized the Wilcoxon rank sum test, a non-parametric statistical test, implemented within the Seurat package. We set stringent criteria to identify significant changes in expression. Only genes or isoforms exhibiting an absolute fold change greater than 2 were considered, ensuring that only biologically meaningful alterations were included in our analysis. Furthermore, to account for multiple testing and reduce the likelihood of false positives, we applied an adjusted *P*-value threshold of less than 0.05.

### Gene ontology enrichment analysis

ClusterProfiler (v4.6.0) (Yu et al., 2012) was used for Gene ontology (GO) enrichment analysis. The first step in our process involved the conversion of our gene collection into recognizable identifiers within established gene databases. For this, “bitr” function was utilized to convert the gene collection into the Entrez gene database and Ensembl database gene number (Entrez ID). “compareCluster” function was then used to achieve functional enrichment terms of the specific genes.

### CNA estimation from long-read scRNA-seq data

We employed the inferCNV tool (Patel et al., 2014) to derive copy number profiles from long-read scRNA-seq data. Large-scale chromosomal copy number variations were analyzed in individual cells from primary tumors, with normal tissue used as a reference to distinguish CNAs from baseline diploid states.

### Calculation of UTR length, UTR deviation and relative differences

We defined a metric, termed “gene UTR deviation”, to quantitatively measure the alterations in 3′- and 5′-UTR length of each gene in individual cells, respectively, using cells from normal epithelium as a baseline for comparison. Firstly, we determined the expressed UTR length (EUL) for each gene in each cell, taking into account the lengths of all identified isoforms. This was accomplished by weighting all isoforms within a gene according to their expression levels. This weighting ensured that the more predominantly expressed isoforms had a proportionally greater influence on the average UTR length, thus reflecting a more accurate representation of the UTR landscape in each cell. Then, the gene UTR deviation (GUD) was done by subtracting the mean expressed UTR length observed in all 374 normal epithelial cells. By doing so, we obtained a deviation value for each gene in each cell. The specific operating formula is as follows:


$$\begin{array}{l} \begin{array}{l} EUL_{g,c}=\frac{\underset{i}{\sum }M_{g,i}\times \left(C_{i,c}\times l_{i}\right)}{\underset{i}{\sum }M_{g,i}\times C_{i,c}}\; \end{array} \end{array}$$ (1)

$$\begin{array}{l} \begin{array}{l} GUD_{g,c}=EUL_{g,c}-\frac{1}{\left|Normal\right|}\underset{c\in Normal}{\sum }\;EUL_{g,c}\; \end{array} \end{array}$$ (2)

where $EUL_{g,c}$ represents the expressed UTR length for gene *g* in cell *c*. $M_{g,i}$ is a binary variable representing the gene (*g*) to isoform (*i*) relationships, with 0/1 indicating the absence/presence of a relationship. $C_{i,c}$ represents the expression of isoforms in cells. $l_{i}$ is the UTR length of isoform *i*. $GUD_{g,c}$ represents the gene UTR deviation for gene *g* in cell *c*.

To identify differential UTR genes between stem/TA-like cells (cancer) and stem/TA cells (normal), we employed a Linear Mixed-Effects Model (LMM) to predict UTR deviation (as defined above) from cell type (stem/TA vs. stem/TA-like), accounting for technological batch (CRC and HTCRC datasets). Genes were classified as having shortened or lengthened UTRs based on the following criteria: (1) The estimate of the cell type coefficient was less than 0 (for UTR shortening) or greater than 0 (for UTR lengthening); (2) The *P*-value associated with the cell type coefficient was less than 0.05; (3) The absolute value of the UTR deviation difference between stem/TA-like and stem/TA cells was greater than 10 bp (for shortening) or less than –10 bp (for lengthening).

### Differential splicing analysis

SUPPA (v2.3) (Trincado et al., 2018) was performed to generate local alternative splicing events of normal epithelial cells and cancer cells respectively. Alternative splicing events were defined into seven patterns: skipping exon (SE), retained exon (RI), alternative 5′ splice-site (A5), alternative 3 ′splice-site (A3), alternative first exon (AF), alternative last exon (AL) and mutually exclusive exons (MX). The differential alternative splicing events were further filtered by retaining events with P-value<0.05 and |ΔPSI|≥
0.1.

### iCMS classification of CRC tumors

Specific marker genes for iCMS2 and iCMS3 subtypes were provided in a previous study (Joanito et al., 2022), including 4 distinct gene sets: upregulated genes and downregulated genes for iCMS2 and iCMS3, respectively. For each gene set, the single-cell module score was computed on log-normalized gene expression matrix, using the AddModuleScore function in Seurat. The final iCMS module scores were computed as the module score of upregulated genes subtracted by that of downregulated genes in each iCMS type. Significant differential expressed transcripts were defined as “Up” or “Down” in an iCMS subtype only if it was consistently upregulated or downregulated relative to both of the other iCMS subtype and normal epithelium, with a fold change ≥ 2 and *P*-value < 0.05. Significant differential alternative splicing events were defined as iCMS2 specific or iCMS3 specific only if it was consistently upregulated relative to both of the other iCMS subtype and normal epithelium, with ΔPSI ≥ 0.1 and *P*-value < 0.05.

### Gene set enrichment analysis

Gene set enrichment analysis (GSEA) was performed using the GSEA software (v4.3.3) (Subramanian et al., 2005). The expression dataset consisted of log_2_(TPM + 1) normalized values, with genes ranked by log_2_(fold change) for enrichment analysis. The Hallmark gene set database (h.all.v2024.1.Hs.symbols.gmt) from the Molecular Signatures Database (MSigDB) was used as the reference gene set. A total of 1000 permutations were conducted to evaluate the statistical significance of each gene set. Significantly enriched pathways were identified based on a false discovery rate (FDR) < 0.05.

### CNA estimation from WES data

Copy number alterations were inferred from whole-exome sequencing (WES) data using CNVkit (v0.9.9) (Talevich et al., 2016). The analysis was performed with tumor samples from colorectal cancer patients and matched normal samples as references. The CNVkit batch command was used to process tumor and normal BAM files from WES data. For visualization, a heatmap of CNV profiles was generated using the heatmap command in CNVkit to compare copy number changes across multiple tumor samples.

### Differential transcript usage analysis

Differential transcript usage (DTU) analysis was performed using the R package DTUrtle (v1.0.2) (Tekath and Dugas, 2021) to compare stem/TA-like cells (cancer) vs. stem/TA cells (normal) in each patient individually. To mitigate batch effects, for each patient in the CRC dataset, stem/TA cells were defined as the combined stem/TA cells from all patients in the CRC cohort. Similarly, for each patient in the HTCRC dataset, stem/TA cells were defined as the combined stem/TA cells from all patients in the HTCRC cohort. DRIMSeq was firstly used for preliminary filtering and differential transcript usage analysis. Then, stageR was utilized to perform statistical test correction. The criteria for defining significant DTU were an overall false discovery rate (OFDR) < 0.05 and the proportion difference in transcript usage for the genes between the two conditions exceeding 10%. An upset plot was generated to visualize the distribution of DTU genes across 12 CRC patients using the ComplexUpset R package. The structures of RNA isoforms were visualized using IsoformSwitchAnalyzeR R package.

### Differential CDS usage analysis

Transcripts were grouped based on their corresponding Consensus Coding Sequence (CCDS) IDs of Ensembl database, generating a CDS expression matrix (CCDS ID × cell). As with the strategy of differential transcript usage analysis, we used DTUrtle package to detected significant differences in the use of CDS regions between stem/TA-like cells (cancer cells) and stem/TA cells (normal epithelial cells) in each patient. The criteria for defining significant DCU were an overall false discovery rate (OFDR) < 0.05 and the proportion difference in CDS usage for the genes exceeding 10%.

### Somatic mutation identification from WES data

Paired-end reads were cleaned by fastp (v0.20.1) and then aligned to the human reference genome GRCh38 using BWA (v0.7.17-r1188). The aligned bam files were subjected to mark duplicates and base quality score recalibration using GATK4 (v4.1.9) with known variation resources (1000G phase1 snps, dbsnp138, mills and 1000G gold standard indels). To identify somatic alterations in colorectal cancer, Mutect2 mode in GATK4 was performed to call SNVs and indels in tumor sample with matched normal sample within individual patient. The short variants were ensured by the following filtering criteria: (1) remaining variants with “PASS” status in “FILTER” column; (2) at least 10× coverage in tumor sample with at least 3× mutation coverage; (3) at least 10× coverage in normal sample with at most 1× mutation coverage. All variants were annotated by ANNOVAR with Ensembl Variant Effect Predictor (VEP).

### Allele specific gene expression analysis

We performed a pileup of each genome-mapping BAM file from individual full-length transcriptome at the somatic mutation sites identified by WES data using the pysam python module. The coverage number of reference and alternative/mutant alleles in individual cells were counted respectively, based on which the following three kinds of matrices were generated: reference/WT allele × cell expression matrix ($E_{wt}$), mutant allele × cell expression matrix ($E_{mut}$) and total × cell expression matrix ($E_{tot}$).

To determine whether a specific mutation identified in WES data exists in individual cells of scRNA-seq, we first calculated the sequencing error rate (ϵ) of a particular allele in individual cells as the ratio of read counts that are neither wild-type nor mutant allele ($C_{other}$):


$$\begin{array}{l} \begin{array}{l} \epsilon =\frac{C_{other}}{C_{wt}+C_{mut}+C_{other}}\; \end{array} \end{array}$$ (3)

Assuming a binomial distribution of read counts and a null hypothesis that the mutant allele was produced by sequencing error, we conducted one-sided binomial test to calculate the *P*-value, testing whether the observed proportion of the mutant allele is significantly greater than the expected error rate. After that, we performed false discovery rate (FDR) correction to adjust the *P*-values obtained from the binomial tests, accounting for multiple testing across all cells and mutations. A mutation was confirmed if FDR<0.05 and $C_{wt}+C_{mut}\geq 3$ in cancer cells or $C_{wt}+C_{mut}\geq 10$ in normal cells.

To explore the transcriptional phenotypic effect of these mutations, we calculated Pearson correlation coefficients between allelic expression matrices of mutation sites ($E_{tot}$, $E_{wt}$, and $E_{mut}$) and gene expression matrix of highly variable genes ($E_{hvg}$) within each patient to produce correlation matrices $R_{tot\cdot hvg}$, $R_{wt\cdot hvg}$, and $R_{mut\cdot hvg}$, respectively.

### siRNA-mediated knockdown of *PPIG* in HCT116 Cells

HCT116 colorectal cancer cells were maintained in Dulbecco’s Modified Eagle Medium (DMEM) supplemented with 10% fetal bovine serum (FBS) and 1% penicillin-streptomycin. Cells were incubated at 37°C in a humidified environment containing 5% CO₂. For siRNA-mediated knockdown of *PPIG*, cells were seeded into 6-well plates at a density of 2 × 10⁵ cells per well. At approximately 80% confluence, cells were transfected using Lipofectamine RNAiMAX (Thermo Fisher Scientific, Cat# 13778150) according to the manufacturer’s instructions.

Three independent siRNAs specifically targeting *PPIG* (si-*PPIG*-1, si-*PPIG*-2, si-*PPIG*-3) were utilized for gene knockdown experiments. Non-targeting control siRNA was included as a negative control (NC). Each siRNA was transfected at a final concentration of 20 nmol/L using Opti-MEM Reduced-Serum Medium (Thermo Fisher Scientific). Cells were incubated for 48 hours post-transfection, after which total RNA was isolated for downstream analysis.

Total RNA was extracted using the RNeasy Mini Kit (QIAGEN) following the manufacturer’s protocol. RNA concentration and integrity were assessed using a NanoDrop spectrophotometer (Thermo Fisher Scientific). cDNA was synthesized using the PrimeScript RT Reagent Kit (Takara), and qRT-PCR was performed with SYBR Green Master Mix (Thermo Fisher Scientific) to confirm knockdown efficiency. GAPDH was used as an internal control. Relative expression levels were calculated using the 2⁻^ΔΔCt^ method.

## Supplementary data

Supplementary data is available at *Protein & Cell* online https://doi.org/10.1093/procel/pwaf049.

pwaf049_suppl_Supplementary_Figures

pwaf049_suppl_Supplementary_Table_1

pwaf049_suppl_Supplementary_Table_2

pwaf049_suppl_Supplementary_Table_3

pwaf049_suppl_Supplementary_Table_4

pwaf049_suppl_Supplementary_Table_5

pwaf049_suppl_Supplementary_Table_6

pwaf049_suppl_Supplementary_Table_7

pwaf049_suppl_Supplementary_Table_8

pwaf049_suppl_Supplementary_Table_9

## Data Availability

The raw sequence data of long-read scRNA-seq and WES data generated in this study have been deposited in the Genome Sequence Archive for Human (GSA-Human) under accession HRA006041. The corresponding processed data are available in the Gene Expression Omnibus (GEO) under accession GSE248094. Publicly available data from the Human Colon Cancer Atlas (c295) were obtained from GEO (GSE178341).

## Code availability

All relevant code for data analysis in this paper is available from the lead contact upon request.

## Artificial intelligence (AI)

No AI-assisted technologies were used in this work.
