# Supplementary material for: Systematic characterization of full-length RNA isoforms in human colorectal cancer at single-cell resolution
Source: Protein Cell. 2025 Jul 22;16(10):873–95. doi: 10.1093/procel/pwaf049 (PMC12578292; doi:10.1093/procel/pwaf049)
Supplement: pwaf049_suppl_Supplementary_Figures [file pwaf049_suppl_supplementary_figures.pdf]

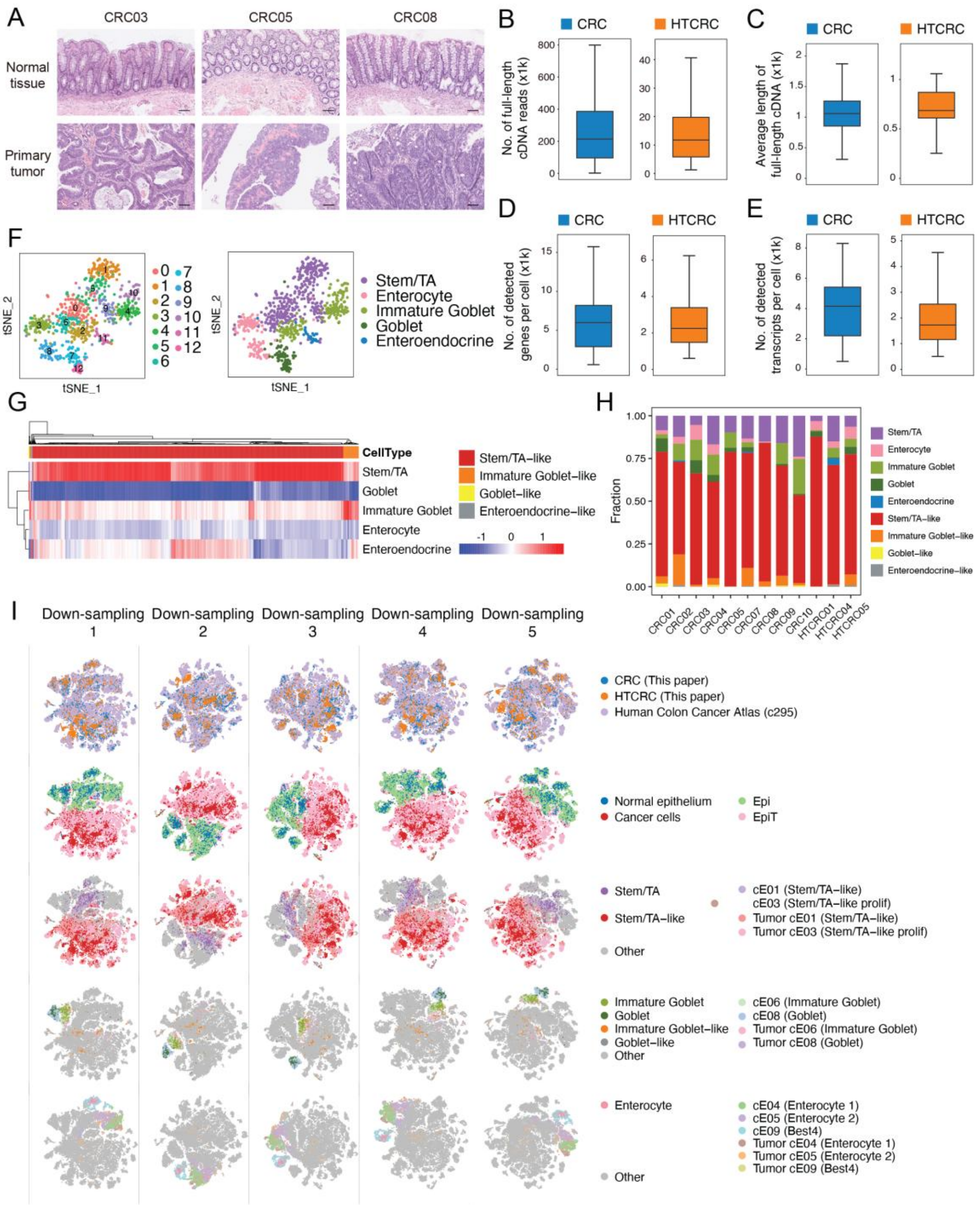

Figure S1. Characterization and validation of cell census of the single-cell full-length transcriptomic atlas in CRC.

(A) Hematoxylin and eosin (H&E) staining of CRC biopsies from representative patients (CRC03, CRC05, CRC08) showing normal and primary tumor tissues. Scale bar: 100 µm. (B–E) Density plots showing (B) the number of full-length cDNA reads per cell, (C) the average length of full-length cDNA reads per cell, (D) the number of detected genes per cell and (E) the number of detected isoforms per cell. (F) t-SNE plots of normal epithelial cells colored by cluster (left) and cell type (right), as detailed in the Methods. (G) RCA clustering of cancer cells projected onto normal epithelial cell types. (H) Bar plot displaying the proportions of annotated cell types in each patient. (I) t-SNE plots showing cells in this study integrated with epithelial cells of the Human Colon Cancer Atlas (c295). The integration was repeated using 5 random splits of the c295 dataset to assess its reliability.

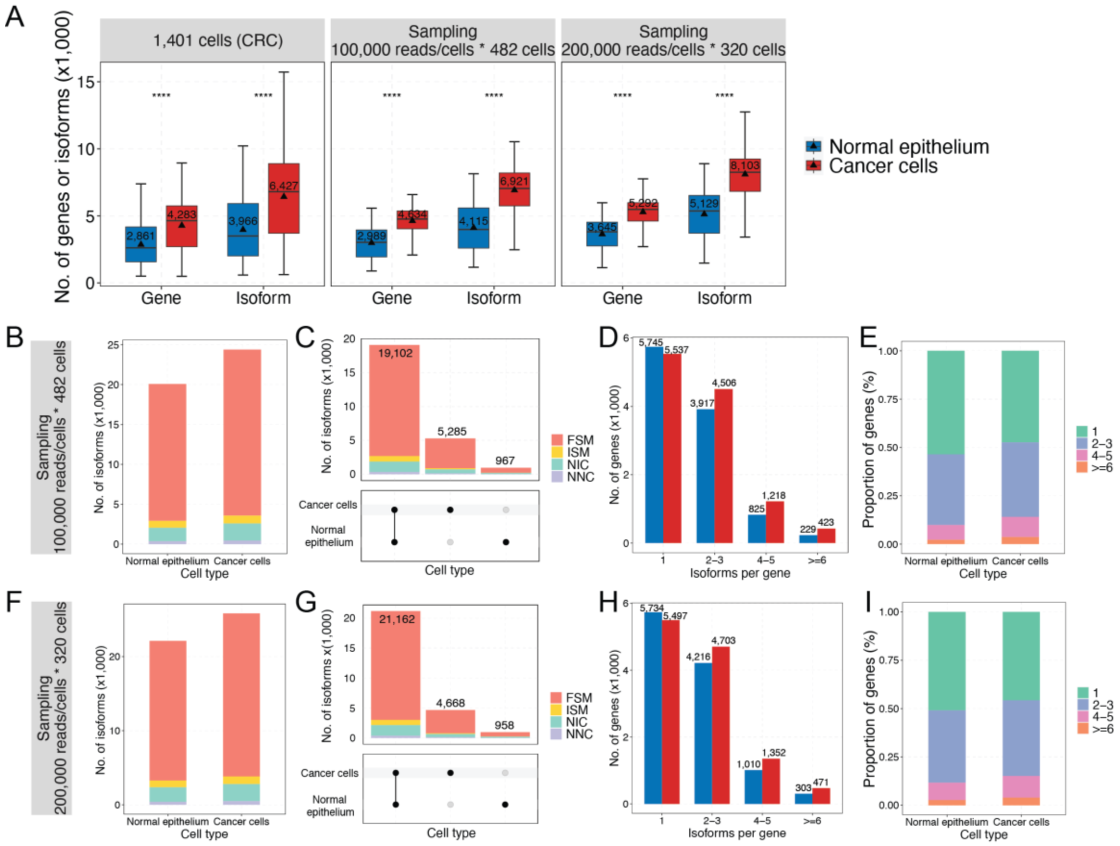

Figure S2. Down-sampling analysis of single-cell full-length transcriptome data.

(A) Boxplot illustrating the number of detected genes and isoforms per cell. Statistical significance was determined using the two-tailed Wilcoxon rank-sum test, with ****$P < 0.0001$. (B–E) Analysis with a down-sampling of 10,000 reads per cell across 482 cells: (B) Bar plot showing the number of isoform categories in normal epithelium and cancer cells; (C) UpSet plot showing the number of transcripts shared between cancer cells and normal epithelium; Bar plot showing (D) the number of and (E) the percentage of genes grouped by the number of expressed isoforms (1, 2–3, 4–5, or ≥6) in normal epithelium and cancer cells. (F–I) Analysis with a down-sampling of 20,000 reads per cell across 320 cells: (F) Bar plot showing the number of isoform categories in normal epithelium and cancer cells; (G) UpSet plot showing the number of transcripts shared between cancer cells and normal epithelium; Bar plot showing the number of (H) and the percentage of (I) genes grouped by the number of expressed isoforms (1, 2–3, 4–5, or ≥6) in normal epithelium and cancer cells.

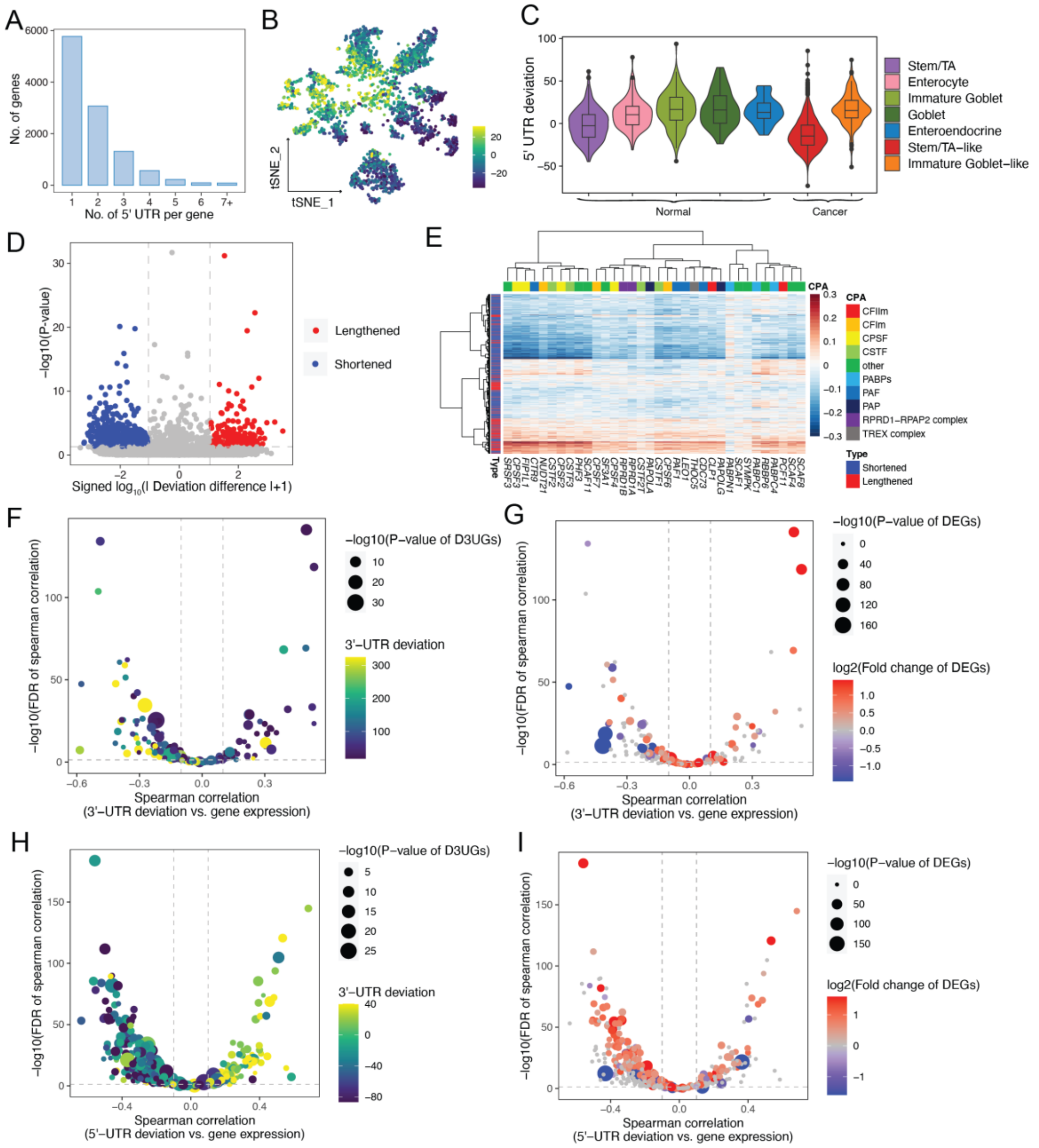

Figure S3. Characterization of 5'-UTR and 3'-UTR lengths in CRC.

(A) Histogram showing distribution of the number of 5'-UTRs per gene. t-SNE visualization of the mean 5'-UTR deviation for all genes across individual cells. The color scale indicates the extent of 5'-UTR length deviation–ranging from shortening (yellow) to elongation (blue). (B) t-SNE visualization of the mean 5'-UTR deviation for all genes across individual cells. Each point represents a single cell, with the color scale indicating the extent of 5'-UTR length deviation (yellow: shortening, blue: elongation). (C) Violin plot of 5'-UTR deviation across different cell types in CRC. (D) Volcano plot of genes with differential 3'-UTR genes between stem/TA-like cells and stem/TA cells. (E) Pearson correlation between 3'-UTR deviation and gene expression of CPA regulators. (F, G) Volcano plots of spearman correlation between 3'-UTR deviation and gene expression in significant 3'-UTR lengthened genes. In panel (F), the dot color represents 3'-UTR deviation difference between stem/TA-like cells and stem/TA cells and the dot size represents FDR value of spearman correlation. In panel (G), the dot color represents the log2 fold change of DEGs between stem/TA-like cells and stem/TA cells and the dot size represents the significance of DEGs. (H, I) Volcano plots of spearman correlation between 5'-UTR deviation and gene expression in significant 5'-UTR shortened and lengthened genes. In panel (H), the dot color represents 5'-UTR deviation difference between stem/TA-like cells and stem/TA cells and the dot size represents FDR value of spearman correlation. In panel (I), the dot color represents the log2 fold change of DEGs between stem/TA-like cells and stem/TA cells and the dot size represents the significance of DEGs.

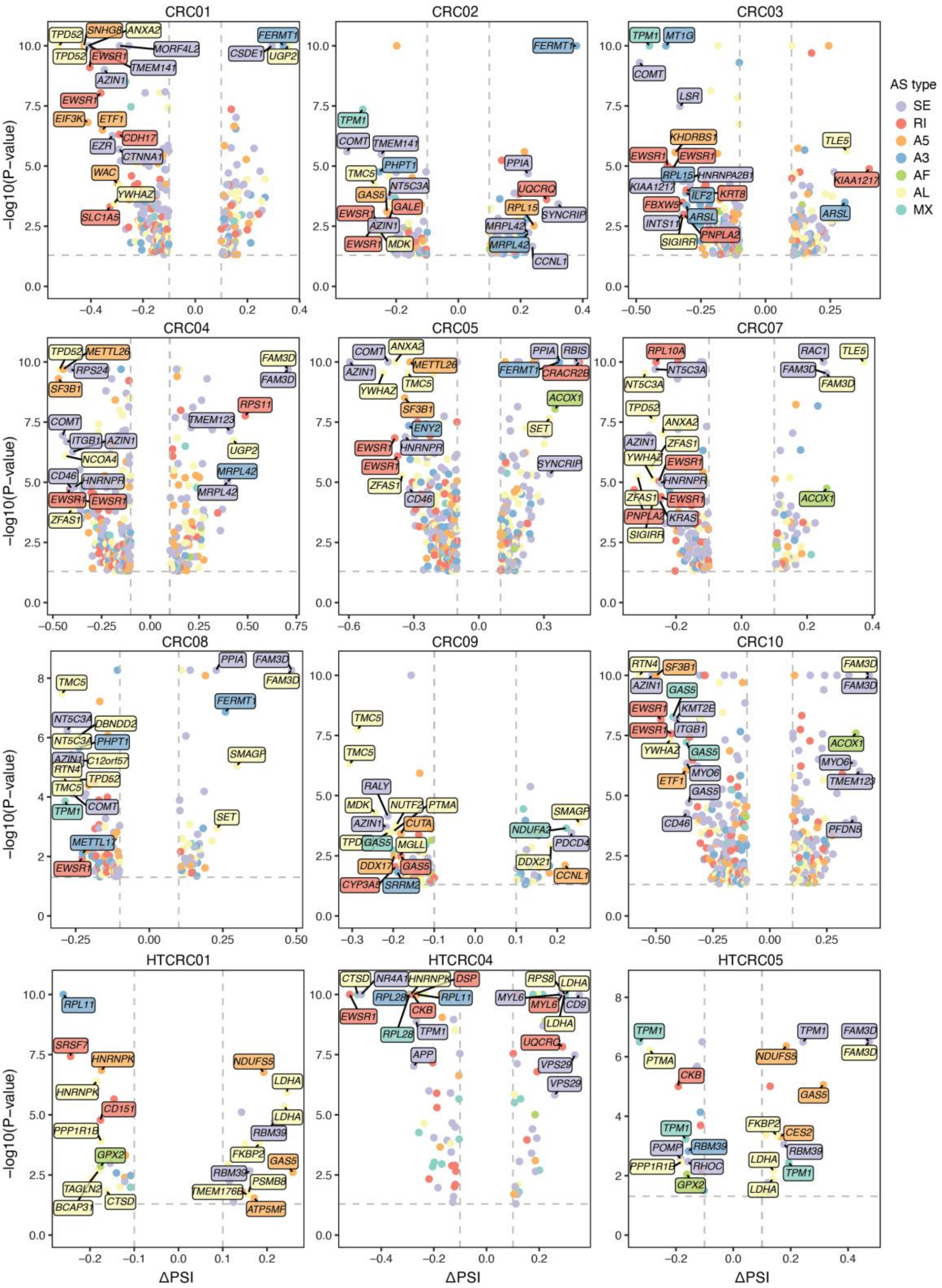

Figure S4. Differential alternative splicing events between stem/TA-like cells and stem/TA cells across patients.

Volcano plots showing differential alternative splicing events between stem/TA-like cells and stem/TA cells in individual patients. Each dot represents an AS event, colored by AS type: skipping exon (SE), retained intron (RI), alternative 5’ splice site (A5), alternative 3’ splice site (A3), alternative first exon (AF), or mutually exclusive exons (MX). The x-axis represents PSI difference (ΔPSI) for stem/TA-like cells versus stem/TA cells, and the y-axis represents the statistical significance. The top 20 genes with the largest ΔPSI differences are annotated on the plots.

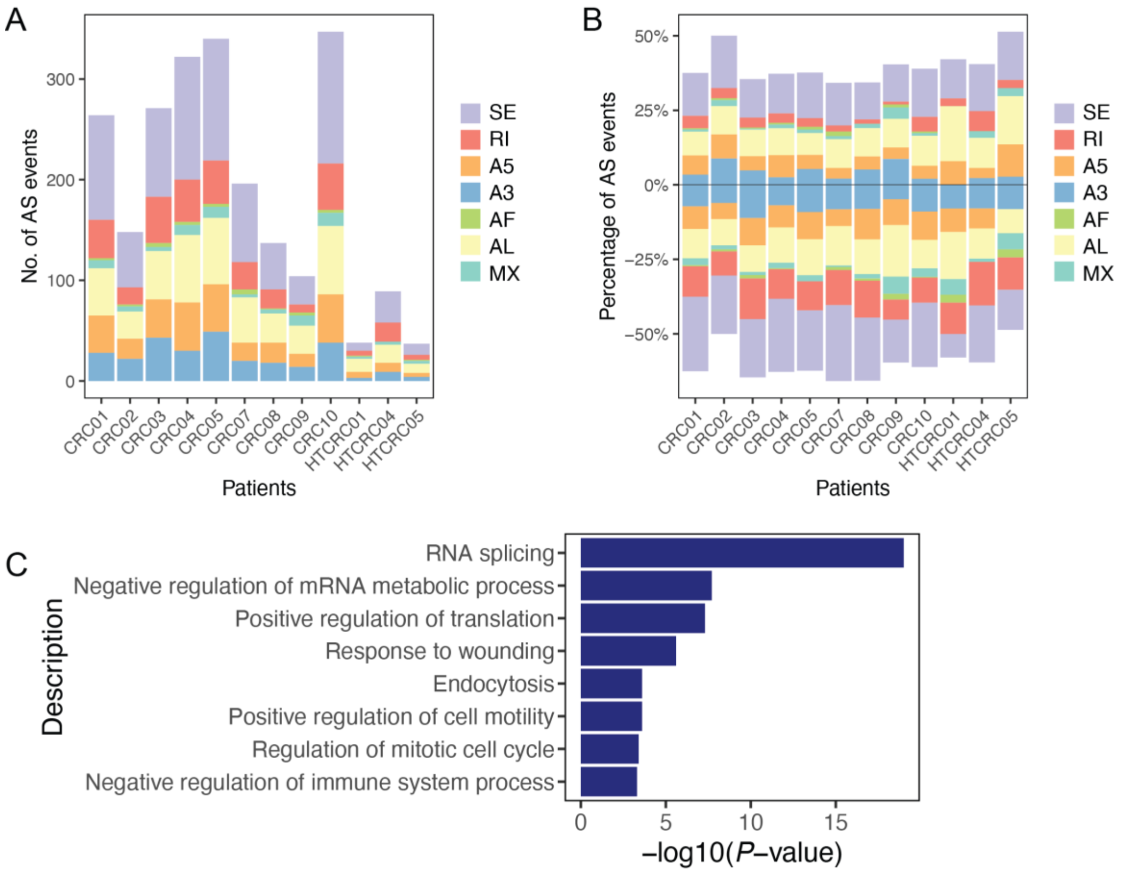

Figure S5. Distribution of differential alternative splicing events between stem/TA-like cells and stem/TA cells across patients.

(A) Stacked bar plot showing the distribution of differential alternative splicing events across individual patients. (B) Stacked bar plot displaying the percentage distribution of differential alternative splicing events in stem/TA-like cancer cells compared to stem/TA cells. Positive and negative values indicate upregulated and downregulated AS events in stem/TA-like cells, respectively. (C) Bar plot illustrating the biological processes enriched among genes with shared AS events (present in at least 3 patients).

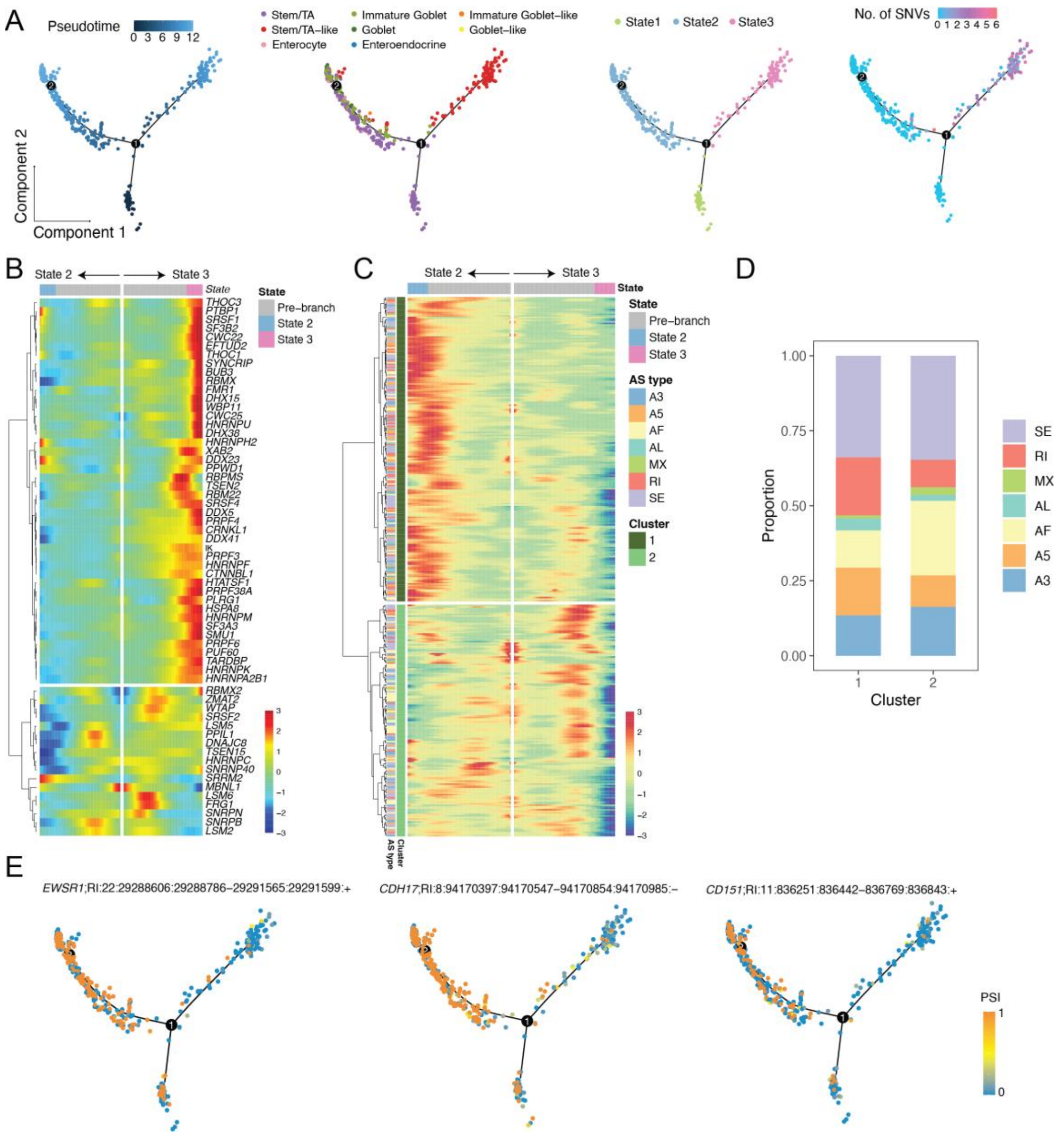

Figure S6. Pseudotime trajectory analysis reveals dynamic alternative splicing events in CRC.

(A) Pseudotime trajectory of epithelial cells from patient CRC01, inferred using Monocle (v2.34.0). Cells are colored by pseudotime (left), cell type (middle left), trajectory state (middle right), and the number of somatic single-nucleotide variants (SNVs) per cell (right). (B) Heatmap of differentially expressed splicing factors across pseudotime states. (C) Heatmap of differential AS events along the pseudotime trajectory. AS events are hierarchically clustered into two major groups, labeled as Cluster 1 and Cluster 2, with associated AS event types indicated. (D) Proportion of AS event types in each cluster. (E) Visualization of retained intron (RI) events in *EWSR1*, *CDH17*, and *CD151* along the pseudotime trajectory. Cells are colored by PSI.

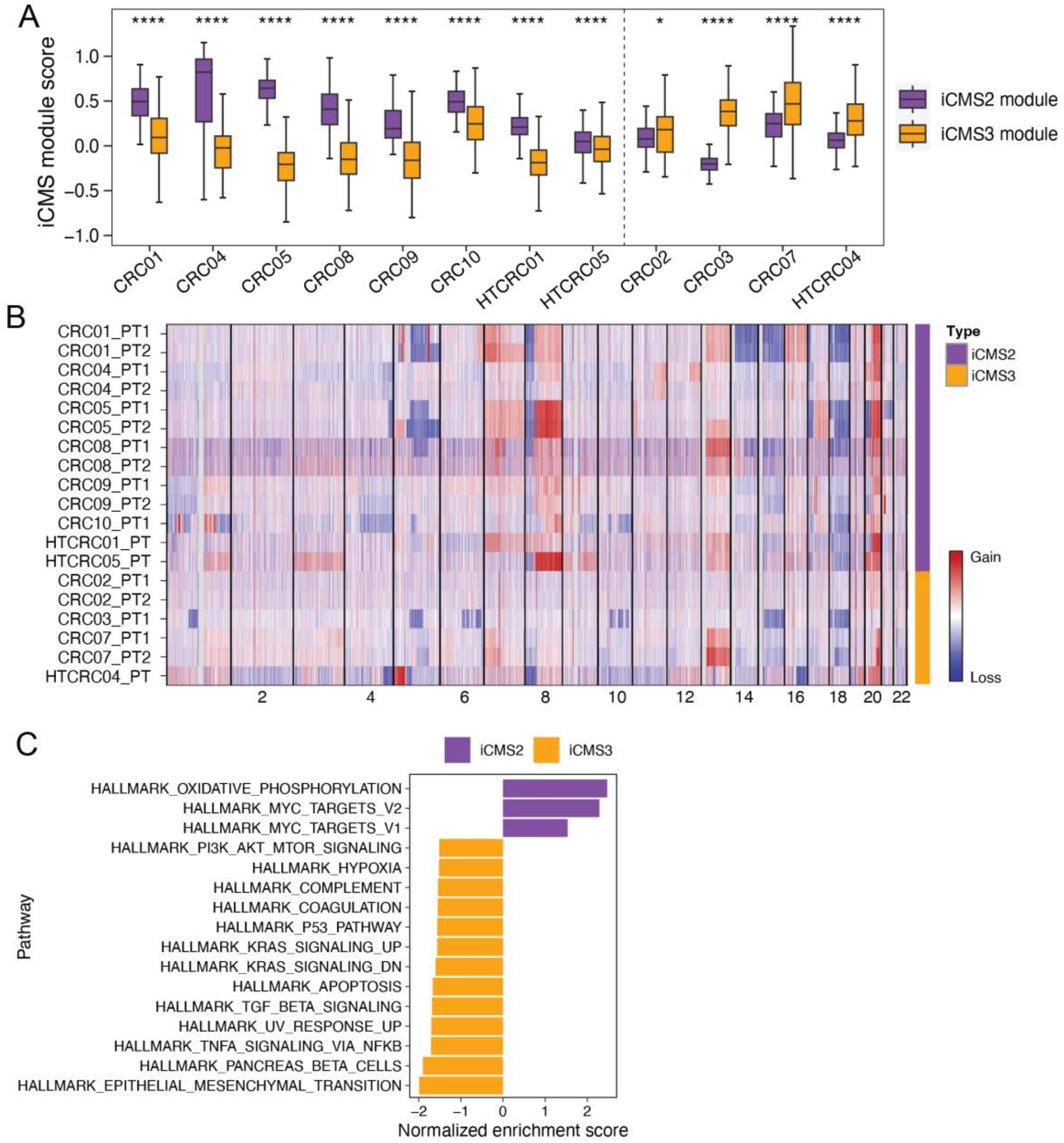

Figure S7. iCMS classification of colorectal cancer in long-read scRNA-seq.

(A) Box plot comparing iCMS module scores between iCMS2 and iCMS3 across CRC patients. Statistical significance was determined using the two-tailed Wilcoxon rank-sum test, with *$p < 0.05$ and ****$p < 0.0001$.

(B) Heatmap displaying copy number alterations (CNAs) across chromosomes for tumor samples derived from WES data. Purple and orange represent iCMS2 and iCMS3 subtypes respectively.

(C) Gene set enrichment analysis (GSEA) of MSigDB hallmark pathways showing normalized enrichment scores (NES) for iCMS2 (purple) and iCMS3 (orange) subtypes.

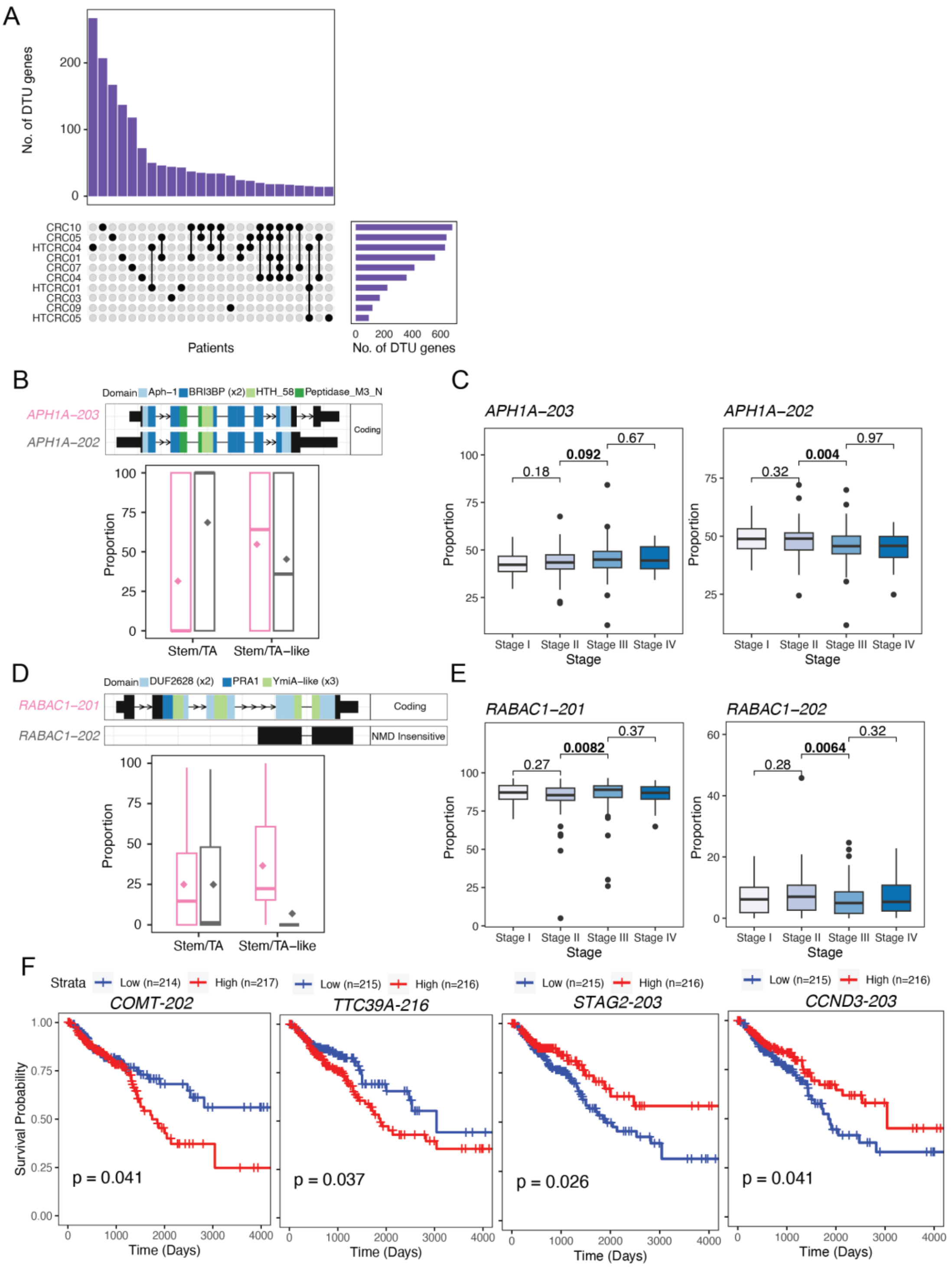

Figure S8. Isoform-level alterations in colorectal cancer progression and prognosis. (A) UpSet plot illustrating the number of DTU genes across individual patients and their overlap. The bar plot at the top represents the number of DTU genes per patient. The intersection matrix below shows shared and unique DTU genes across patients. The inset on the right displays the total number of DTU genes across all patients. (B) Schematic of *APH1A* isoforms (top) and box plots showing the proportion of *APH1A-203* and *APH1A-202* isoforms in stem/TA and stem/TA-like cells (bottom). (C) Box plots show the proportion of *APH1A-203* and *APH1A-202* isoforms across colon adenocarcinoma (COAD) and rectum adenocarcinoma (READ) tumor stages in the TCGA cohort. Statistical significance was assessed using the Wilcoxon rank-sum test. (D) Schematic of *RABAC1* isoforms (top) and box plots showing the proportion of *RABAC1-201* and *RABAC1-202* isoforms in stem/TA and stem/TA-like cells (bottom). (E) Box plots show the proportion of *RABAC1-201* and *RABAC1-202* isoforms across COAD and READ tumor stages in the TCGA cohort. Statistical significance was assessed using the Wilcoxon rank-sum test. (F) Kaplan-Meier survival curves of representative isoforms whose altered proportions are significantly associated with patient survival

in COAD and READ datasets from the TCGA cohort.

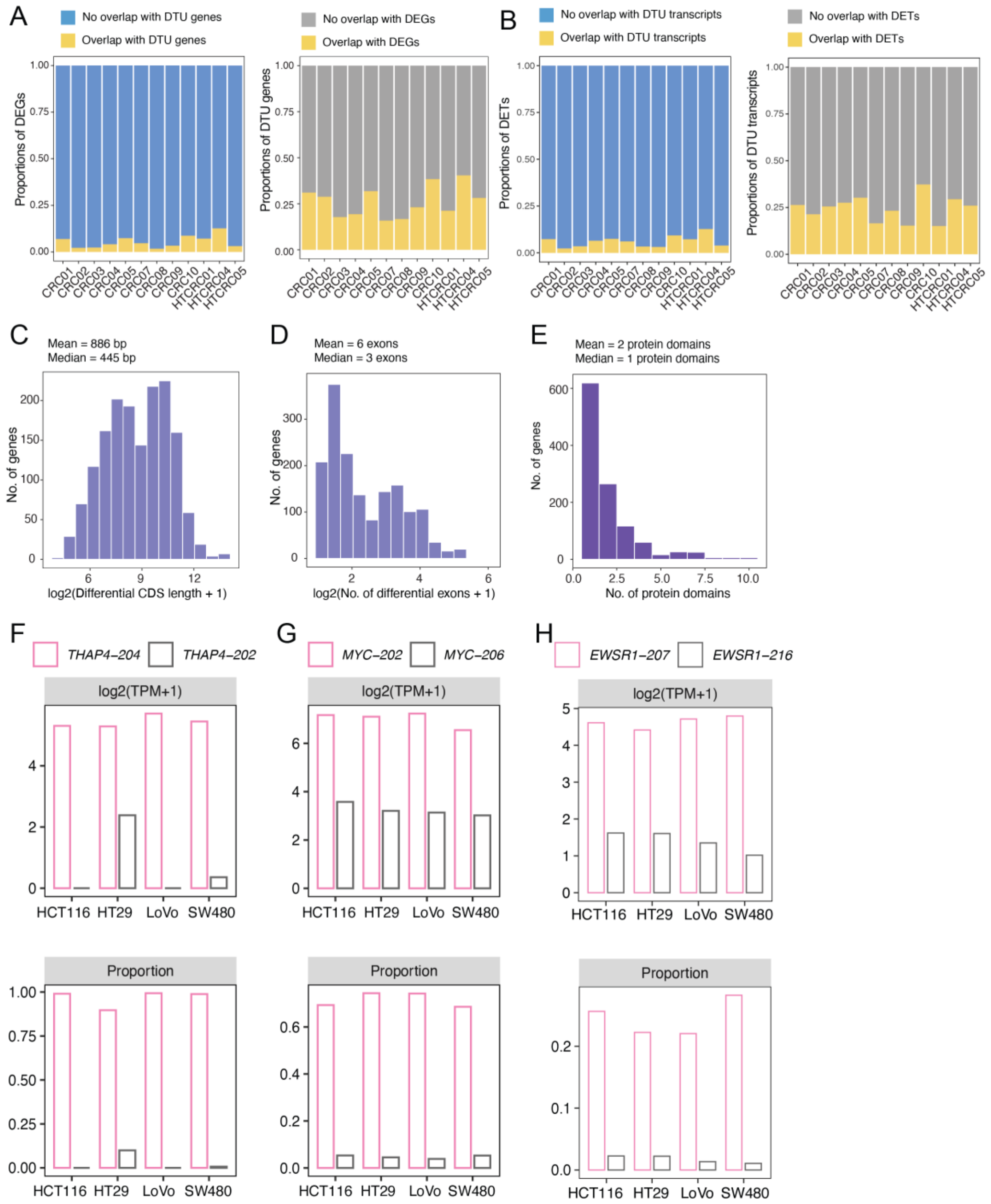

Figure S9. Characterization of Differential Transcript Usage (DTU) and Its Functional Impact in CRC.

(A) Bar plot showing the proportions of DEGs overlapping (yellow) and non-overlapping (blue) with DTU genes (left); and the proportions of DTU genes overlapping (yellow) and non-overlapping (gray) with DEGs (right). (B) Bar plot showing the proportions of DETs overlapping (yellow) and non-overlapping (blue) with DTU transcripts (left); and the proportions of DTU transcripts overlapping (yellow) and non-overlapping (gray) with DETs (right). (C) Histogram showing the distribution of differential CDS lengths among DTU genes, with the mean and median lengths indicated. (D) Histogram showing the number of differential exons among DTU genes, with the mean and median exon counts indicated. (E) Histogram showing the number of protein domains impacted by DTU events, with the mean and median domain counts indicated. (F) Bar plots depicting the absolute expression (log2(TPM+1)) and relative proportions of isoforms for *THAP4*, *MYC*, and *EWSR1* in different CRC cell lines (HCT116, HT29, LoVo, SW480).

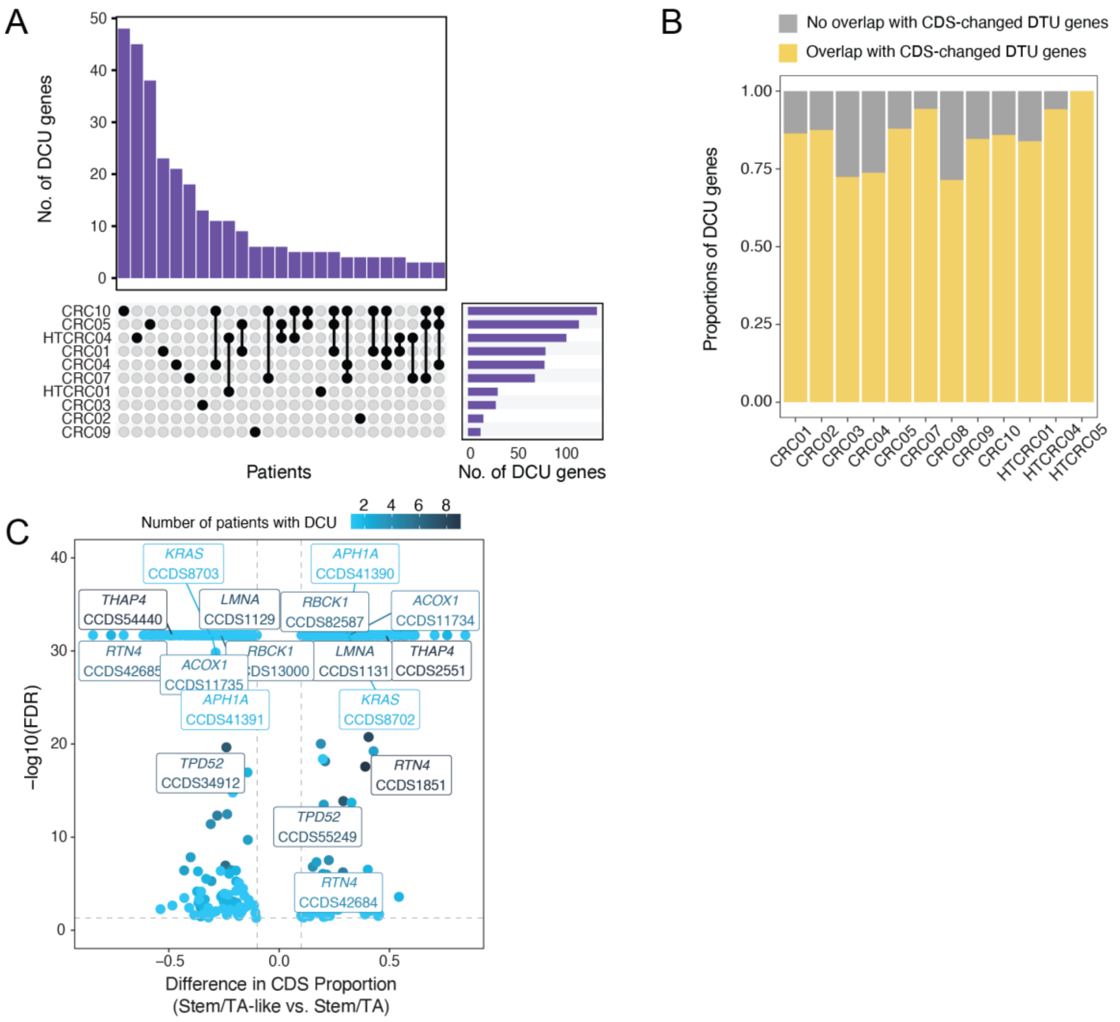

Figure S10. Differential CDS usage characteristics in CRC.

(A) UpSet plot illustrating the number of DCU genes across individual patients and their overlap. The bar plot at the top represents the number of DCU genes per patient. The intersection matrix below shows shared and unique DCU genes across patients. The inset on the right displays the total number of DCU genes across all patients. (B) Bar plot showing the proportion of DCU genes overlapping (yellow) and non-overlapping (gray) with CDS-changed DTU genes. (C) Volcano plot showing differential CDS usage between stem/TA-like cells and stem/TA cells. Each dot represents a CDS, with color indicating the number of patients in which the DCU is observed. The x-axis indicating the average proportion difference of DCU genes across corresponding patients, and the y-axis showing the minimum statistical significance (-$\log_{10}$FDR) among those patients. Key recurrent genes are highlighted and annotated with their respective CCDS identifiers.

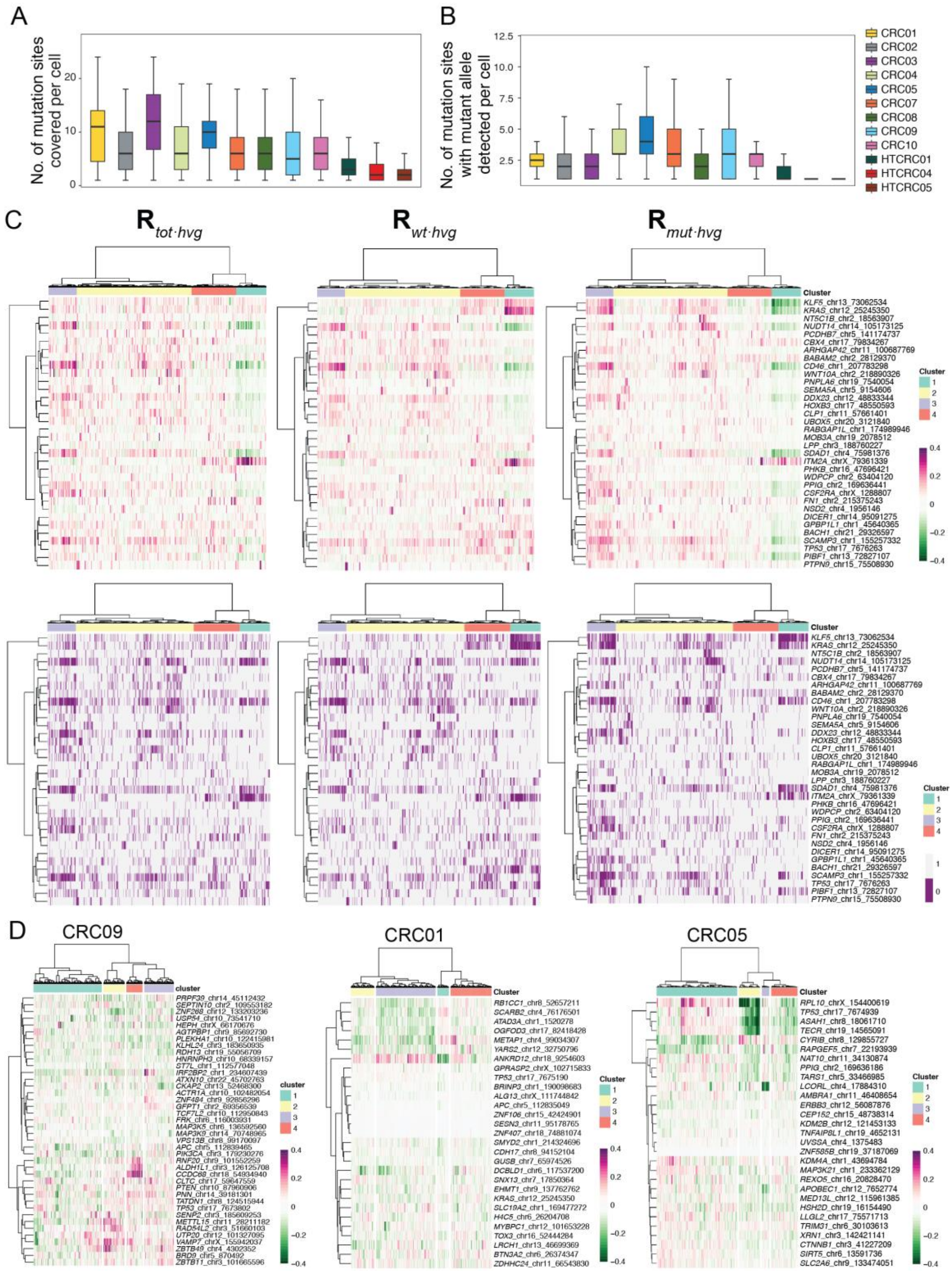

Figure S11. Pearson correlation analysis between allelic expression of mutation sites and highly variable gene expression.

(A) Boxplot showing the number of mutation sites covered per cell across patients. (B) Boxplot showing the number of mutation sites with mutant allele detected per cell across patients. (C) Heatmap of Pearson correlation matrices (top) and significance (bottom) between allelic expression matrices of mutation sites (E_{tot}, E_{wt} and E_{mut}) and gene expression matrix of highly variable genes (E_{hvg}), respectively, in patient CRC07. The presentation of the cluster dendrogram is unified according to the differential correlation matrix ($\mathrm{R}_{mut\cdot hvg} - \mathrm{R}_{wt\cdot hvg}$). (D) Heatmap of the correlation difference between $\mathrm{R}_{mut\cdot hvg}$ and $\mathrm{R}_{wt\cdot hvg}$ in patient CRC09 (left), CRC01 (middle), CRC05 (right).

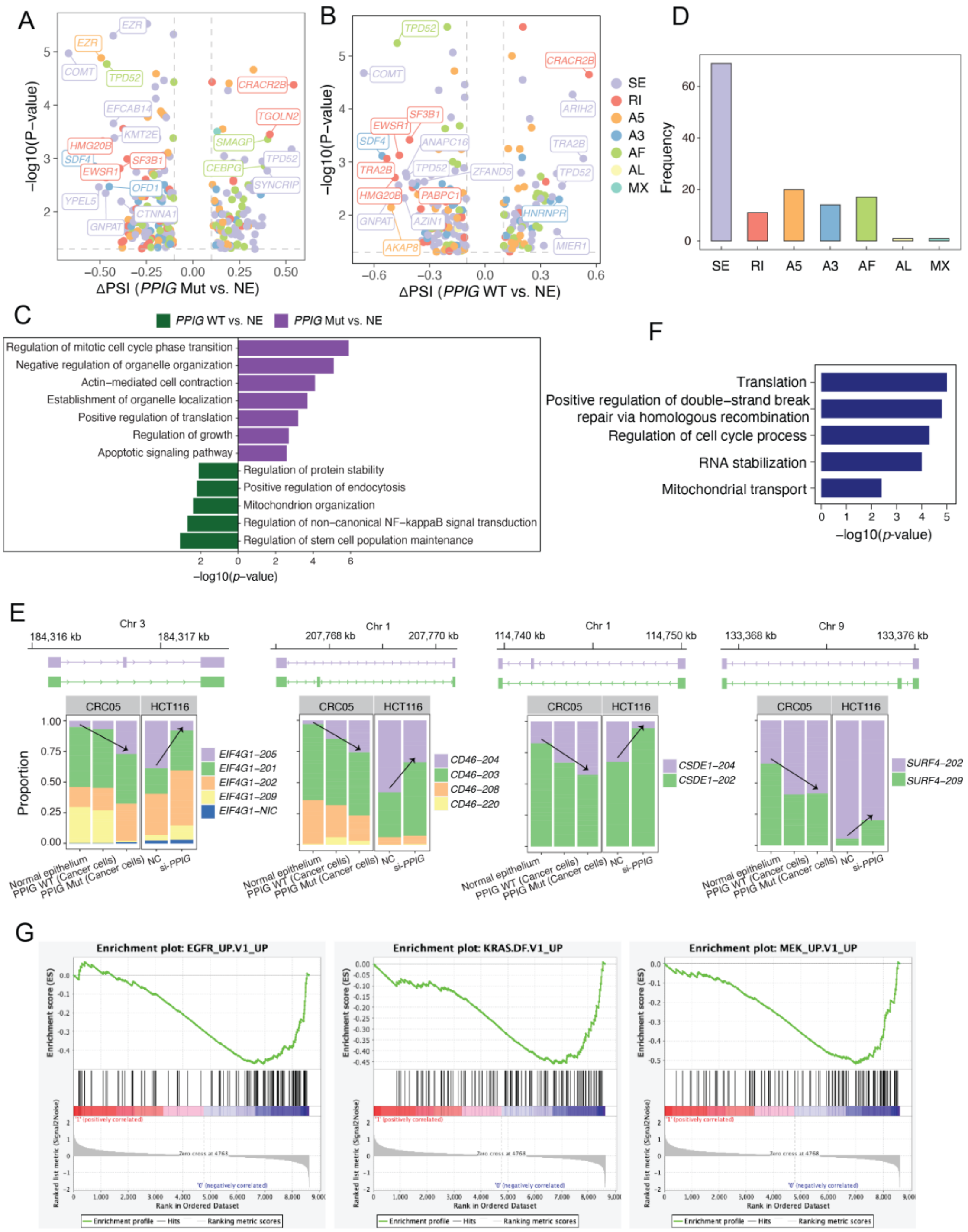

Figure S12. Functional impact of *PPIG* knockdown on alternative splicing in colorectal cancer.

(A) Differential alternative splicing events for *PPIG* mutant cancer cells versus normal epithelium. The top 20 genes with the largest ΔPSI differences are annotated on the plots. (B) Differential alternative splicing events for *PPIG* wild-type cancer cells versus normal epithelium. The top 20 genes with the largest ΔPSI differences are annotated on the plots. (C) Bar plot of GO terms enriched for differential alternative splicing genes between *PPIG* WT cells versus normal epithelial cells (green) and *PPIG* mutant cells versus normal epithelial cells (purple). (D) Frequency distribution of differential AS event types. SE, skipping exon, RI, retained intron, A5, alternative 5' splice site, A3, alternative 3' splice site, AF, alternative first exon, AL, alternative last exon, MX, mutually exclusive exons. (E) Stacked bar plots showing the proportion of isoform expression for *CTNNA1*, *CD46*, *CSDE1* and *SURF4* in long-read scRNA-seq data from CRC05 and long-read bulk RNA-seq data from HCT116 cells. Arrows indicate the direction of PSI changes. (F) Gene ontology enrichment analysis of the 133 differentially spliced genes between *PPIG* knockdown and NC groups. (G) Enrichment plots of gene set enrichment analysis between *PPIG* knockdown and NC groups.

Table S1. Clinical information and cell number distribution of 12 colorectal cancer patients.

Table S2. Quality assessment and cell type annotation of single-cell full-length transcriptomic data from colorectal cancer patients.

Table S3. Structure annotation result of 29,429 isoforms.

Table S4. Differential 3'-UTR deviation genes and 5'-UTR deviation genes between stem/TA-like cells (cancer) and stem/TA cells (normal).

Table S5. Differential alternative splicing events between stem/TA-like cells (cancer) and stem/TA cells (normal).

Table S6. Differential alternative splicing events between iCMS2 and iCMS3 subtypes.

Table S7. Differential transcript usage between stem/TA-like cells (cancer) and stem/TA cells (normal).

Table S8. Differential CDS usage between stem/TA-like cells (cancer) and stem/TA cells (normal).

Table S9. Somatic mutation landscape of the 12 colorectal cancer patients.
